# Targeting RIP Kinases in Chronic Inflammatory Disease

**DOI:** 10.3390/biom11050646

**Published:** 2021-04-28

**Authors:** Mary Speir, Tirta M. Djajawi, Stephanie A. Conos, Hazel Tye, Kate E. Lawlor

**Affiliations:** 1 Centre for Innate Immunity and Infectious Diseases, Hudson Institute of Medical Research, Clayton, VIC 3168, Australia; Mary.speir@hudson.org.au (M.S.); Tirta.Djajawi@hudson.org.au (T.M.D.); Stephanie.conos@hudson.org.au (S.A.C.); Hazel.Tye@hudson.org.au (H.T.); 2 Department of Molecular and Translational Science, Monash University, Clayton, VIC 3168, Australia

**Keywords:** apoptosis, necroptosis, RIP kinases, chronic inflammatory disease, tumour necrosis factor, interleukin-1

## Abstract

Chronic inflammatory disorders are characterised by aberrant and exaggerated inflammatory immune cell responses. Modes of extrinsic cell death, apoptosis and necroptosis, have now been shown to be potent drivers of deleterious inflammation, and mutations in core repressors of these pathways underlie many autoinflammatory disorders. The receptor-interacting protein (RIP) kinases, RIPK1 and RIPK3, are integral players in extrinsic cell death signalling by regulating the production of pro-inflammatory cytokines, such as tumour necrosis factor (TNF), and coordinating the activation of the NOD-like receptor protein 3 (NLRP3) inflammasome, which underpin pathological inflammation in numerous chronic inflammatory disorders. In this review, we firstly give an overview of the inflammatory cell death pathways regulated by RIPK1 and RIPK3. We then discuss how dysregulated signalling along these pathways can contribute to chronic inflammatory disorders of the joints, skin, and gastrointestinal tract, and discuss the emerging evidence for targeting these RIP kinases in the clinic.

## 1. Introduction

Common chronic inflammatory and autoinflammatory diseases are caused by immune dysregulation, the aetiology of which involves a complex interplay between genetic and environmental factors that is incompletely understood [1]. Unmistakably, however, the underlying cause of distinct tissue pathologies is a failure to resolve inflammation arising from the excessive production of inflammatory cytokines and chemokines, as well as danger-associated molecular patterns (DAMPs) that are released from dying cells. Tumour necrosis factor (TNF) is the archetypal death ligand and a pivotal pathogenic cytokine in many common inflammatory diseases. Accordingly, a number of TNF antagonists, as well as other anti-inflammatory biological agents that dampen its activity, have met with considerable success in the clinic. Yet, many patients still fail to respond, or else develop severe adverse reactions to these therapies, highlighting the need for a both a greater understanding of TNF signalling on a cell-by-cell level and also the need for alternative strategies to target TNF signalling pathways in disease.

Over the last decade, the serine-threonine receptor-interacting protein (RIP) kinases -1 and -3 have emerged as key regulators of innate immunity via their integral roles in cell death signalling during cellular stress and following exposure to inflammatory and infectious stimuli [2,3,4]. RIPK1 has a critical scaffolding role in tumour necrosis factor receptor-1 (TNFR1) and Toll-like receptor 3/4 (TLR3/4) pro-inflammatory signalling [5,6,7], as well as a kinase-dependent role in both apoptotic and necroptotic cell death [8]. The carboxy-terminal death domain (DD) of RIPK1 can facilitate its interaction with other DD-containing proteins to promote formation of death receptor signalling complexes [9,10,11], while its RIP homotypic interaction motif (RHIM) domain (also shared with RIPK3) enables RIPK1/3 interactions with other cell death and/or immune adaptors [12], e.g., TIR domain-containing adaptor-inducing IFN-β (TRIF) [5,13] and the dsDNA receptor Z-DNA binding protein-1 (ZBP1; also known as DAI) [14].

**Figure 1:**
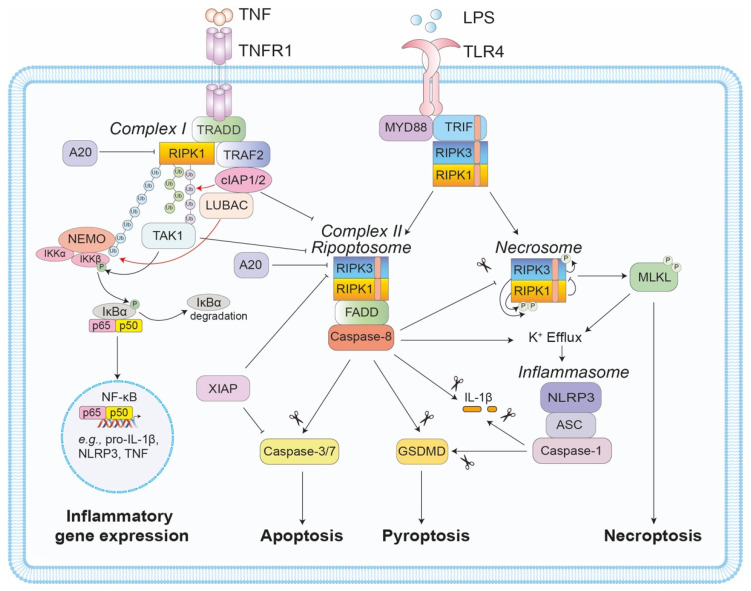
Regulation of RIPK1- and RIPK3-mediated cell death and inflammation. TNF ligation of its receptor, TNFR1, typically induces complex 1 formation, where TNFR1 associates with RIPK1 and TRADD. TRADD subsequently interacts with TRAF2 to facilitate cIAP1 and LUBAC recruitment to the complex. The cIAPs and LUBAC ubiquitylate RIPK1 to facilitate a signalling cascade that induces pro-survival and pro-inflammatory gene expression. If either RIPK1 phosphorylation or ubiquitylation are impaired, FADD-RIPK1(RIPK3)-caspase-8 associate to form the pro-apoptotic complex II (or the ripoptosome). If caspase-8 activity is compromised, a further complex, termed the necrosome, forms in a RIPK1- and RIPK3 kinase-dependent manner. Within this complex, RIPK3-mediated phosphorylation and activation of MLKL induces necroptosis, a lytic inflammatory form of cell death. Of note, TLR4 ligation can also induce similar inflammatory (e.g., RIPK3-caspase-8-mediated cytokine transcription) and cell death events via RHIM-RHIM interactions between the adaptor TRIF and RIPK1/3. In addition to cell death and cytokine transcription, it is now appreciated that both the ripoptosome and the necrosome can promote inflammasome assembly, i.e., caspase-8 and MLKL activity can trigger NLRP3 inflammasome activation, via K^+^ efflux, while caspase-8 can also directly cleave IL-1β and GSDMD to induce inflammation.

TNF ligation of TNFR1 typically recruits RIPK1 into a membrane bound receptor signalling complex, referred to as complex I, that activates NF-κB-mediated transcription of pro-survival genes (Figure 1). Along with RIPK1, TNFR1-associated death domain protein (TRADD) is recruited to TNFR1 via their common DD [15,16]. The adaptor TNFR-associated factor 2 (TRAF2) subsequently binds to TRADD and recruits the cellular inhibitor of apoptosis proteins (cIAP)-1 and cIAP2 [17,18]. The E3 ubiquitin ligase activity of cIAP1/2 attaches K63- and K11-linked polyubiquitin chains to RIPK1, allowing for the recruitment of the linear ubiquitin chain assembly complex (LUBAC), which is a heterotrimeric complex made up of haeme-oxidized IRP2 ubiquitin ligase 1 (HOIL-1), HOIL-1-interacting protein (HOIP), and SHANK-associated RH domain-interacting protein (SHARPIN). Subsequent M1-linked ubiquitylation of complex I components by LUBAC stabilises the complex and promotes the TAK1- and IKK-dependent activation of canonical NF-κB transcription [19,20,21]. Post-translational modification of RIPK1 within complex I, as well as the selective cleavage of RIPK1 and RIPK3 (and cylindromatosis [CYLD]) by caspase-8, maintain TNF-mediated pro-survival signalling and block the transition to pro-apoptotic cell death complex II [22,23,24,25,26,27,28,29]. This critical pro-survival scaffolding role for RIPK1 is evidenced by the fact that RIPK1-deficient mice die shortly after birth due to systemic multi-organ inflammation mediated by unrestrained apoptosis and necroptosis [30,31,32,33,34,35,36].

Complex II, and the related ripoptosome complex that can form in the absence of death receptor ligation [37,38], are essentially comprised of the adaptor protein Fas-associated protein with death domain (FADD), RIPK1, RIPK3, and pro-caspase-8 (Figure 1). Known triggers of complex II/ripoptosome include cIAP loss, via the natural IAP antagonist second mitochondria-derived activator of caspases (Smac; also known as DIABLO), or genetic/chemical depletion [25,37,38,39,40], as well as loss/inhibition of TRAF2, TAK1, or IKK-β/γ [18,29,41]. Once formed, catalytically active caspase-8 within complex II triggers apoptosis by cleaving and activating the downstream effector caspases-3 and -7. Caspase-8 can also cleave and truncate Bid to engage the mitochondrial apoptosis pathway and amplify death in certain cell types (i.e., type II cells, including hepatocytes) [42,43,44]. It is worth mentioning, that while antagonism of X-linked IAP (XIAP) by Smac is thought to promote apoptosis indirectly by unleashing caspase-3, -7, and -9 activity, the ubiquitin E3 ligase activity of XIAP also critically represses TNFR1 and TLR3/4-induced cell death in myeloid cells [45,46,47,48,49].

When the activity of caspase-8 is compromised, either genetically or by viral/chemical inhibition, both TNFR1 and TLR-TRIF signalling can trigger a RIP kinase-dependent, caspase-independent form of lytic cell death called necroptosis (Figure 1) [50,51]. In the case of TNF signalling, loss of both the IAPs and caspase-8 activity promotes full-length RIPK1 and RIPK3 interactions via their RHIM domains to form a functional oligomeric amyloid structure, termed the necrosome [52]. Next, autophosphorylation of RIPK3 facilitates the recruitment of the terminal necroptotic effector, the pseudokinase mixed-lineage kinase domain-like (MLKL). Subsequent phosphorylation of the MLKL activation loop by RIPK3 triggers a conformational change that exposes its N-terminal 4-helix bundle (4HB) killing domain and promotes the assembly of pore-forming oligomers within the plasma membrane and the lytic release of the cellular contents, including inflammatory DAMPs, e.g., interleukin (IL)-1α, IL-33, high-mobility group box 1 (HMGB1), ATP, uric acid, and heat shock proteins (HSPs) [53,54,55,56,57,58]. Of note, while the kinase activity of RIPK1 is essential for TNF-mediated necroptosis, it is not required for TLR-TRIF signalling. Moreover, RIPK1 inhibits the necroptotic activity of RIPK3 in response to TLR-TRIF signalling [45], and its RHIM domain also acts as a brake on ZBP1-RIPK3-mediated necroptosis during development, via an unknown mechanism [59,60,61].

It has recently emerged that significant crosstalk exists between extrinsic cell death and inflammatory signalling, particularly with regard to the NOD-like receptor protein 3 (NLRP3) inflammasome (reviewed in [42,62]). The multimeric cytosolic NLRP3 inflammasome complex comprises the sensor protein NLRP3 that is triggered by a number of host and pathogen danger molecules and instigates stepwise inflammasome assembly via recruitment of the adaptor protein ASC (apoptosis-associated speck-like protein containing a CARD) and pro-caspase-1. Active caspase-1 then cleaves pro-IL-1β and pro-IL-18 into their mature bioactive forms and activates the pore-forming effector, gasdermin D (GSDMD), to induce pyroptotic cell death. A number of studies have now highlighted how cell death machinery can regulate NLRP3 inflammasome activity via effects on transcriptional responses and post-translational events that facilitate complex assembly [63]. For example, TLR-induced NLRP3 inflammasome priming (i.e., induction of core inflammasome machinery NLRP3 and pro-IL-1β) and post-translational activity was suggested to be dependent on a FADD-caspase-8 complex in response to both canonical and non-canonical caspase-11 inflammasome stimuli [64]. Whilst the defective transcriptional pathway associated specifically with blunted enzymatic caspase-8 activity upon TLR ligation in *Ripk3^−/−^Caspase-8^−/−^*, *Ripk3^−/−^Fadd^−/−^*, and *Fadd^−/−^Mlkl^−/−^* bone marrow-derived macrophages (BMDM) and bone marrow-derived dendritic cells (BMDC) remains controversial [64,65,66,67,68], it is now appreciated that a FADD-RIPK1-caspase-8 scaffolding complex supports inflammasome responses [69,70], and that caspase-8 can also interact with ASC to coordinate cell death and IL-1β activation (in the absence of caspase-1) [71,72,73].

It is now also becoming increasingly clear that RIPK1 and RIPK3 can coordinate alternative and cell death-induced pathways to NLRP3 inflammasome and IL-1β activation [42,74,75] (Figure 1). Loss of key negative regulators of cell death, such as the IAPs, the NF-κB inhibitor and deubiquitinase A20, and TAK1, which promotes phosphorylation events that prevent RIPK1 death signalling [76,77], can also induce modes of RIPK1 (RIPK3)-FADD-caspase-8-mediated NLRP3 inflammasome activation [45,46,47,49,78,79,80,81,82,83,84,85]. For example, the potassium efflux (K^+^) associated with necroptotic cell death in IAP- and/or caspase-8-deficient cells can potently activate the NLRP3 inflammasome in a cell intrinsic manner [45,86,87]. Likewise, RIPK1 can act as a repressor of RIPK3/MLKL-mediated necroptotic activation of NLRP3 [45]. Additionally, TLR4-TRIF (or TNFR) signalling in IAP-depleted myeloid cells can induce the direct caspase-8-mediated proteolysis of IL-1β, as well as NLRP3 inflammasome activation that is dependent on K^+^ efflux linked to caspase-8-mediated apoptosis occurring upstream [45,78,88].

TNFR1 signalling via RIPK1 has been long recognised for its role in driving pathogenic pro-inflammatory cytokine and chemokine production in common diseases, such as Rheumatoid arthritis (RA), psoriasis, ankylosing spondylitis, and inflammatory bowel disease (IBD). Hence, TNF antagonists with different mechanisms of action have been used extensively, and with considerable success, to treat these disorders [89,90,91]. Intriguingly, recent evidence shows that pathology in a number of rare autoinflammatory syndromes is driven by excessive RIPK1/3-mediated cell death and inflammasome-associated IL-1β and IL-18 production [92]. Consequently, RIP kinase inhibitors have significant therapeutic potential beyond simply limiting TNF-induced inflammation. To date, small-molecule inhibitor development has preferentially focused on targeting the kinase activity of RIPK1 [93,94], as RIPK3 kinase inhibitor development has been thwarted by the fact they can promote apoptosis via a RIPK1-FADD-caspase-8-cFLIP complex. This phenomenon is analogous to the embryonic lethality observed in RIPK3 kinase dead (*Ripk3*^D161N/D161N^) mice [95,96]. Since knock-in mice expressing a kinase dead form of RIPK1 are healthy [96,97], it is predicted that inhibitors targeting the kinase activity of RIPK1 will be well tolerated and not disrupt its critical pro-survival scaffolding role. It is also worth acknowledging that, unlike RIPK1-deficient mice that die perinatally, patients with biallelic loss-of-function mutations in RIPK1 survive into early adulthood but do develop primary immunodeficiency typified by recurrent infections, as well as clinical manifestations including oral/skin lesions, arthritis, and early-onset IBD [98,99]. This partial redundancy in humans again highlights RIPK1 as a suitable druggable target for disease.

Current efforts to generate RIPK1 inhibitors have yielded small molecules that bind within the hydrophobic pocket of RIPK1, located between the N- and C-terminus of the kinase domain, and stabilise RIPK1 in an inactive conformation [100]. Pre-clinical studies using RIPK1 kinase dead mice and the first generation indole-hydantoin RIP1 kinase inhibitors, Necrostatin-1s (Nec-1s), and less-specific analogue Nec-1, have demonstrated the potential to target RIPK1 signalling in numerous injury (e.g., renal ischaemic reperfusion, hypoxic brain injury), acute (e.g., systemic inflammatory response syndrome), and chronic inflammatory disease models (e.g., multiple sclerosis) (comprehensively reviewed in [42,94,101]). Now, a number of more specific, small-molecule RIPK1 inhibitors have been developed by Glaxo-Smith Kline (GSK), Sanofi/Denali (DNL), Takeda, and Genentech, which are either undergoing pre-clinical testing or have entered early-phase clinical trials, including the benzoxazepinone GSK2982772 for the treatment of psoriasis, ulcerative colitis (UC), and RA, and brain-penetrant DNL747 for the treatment of the neurodegenerative disease Amyotrophic Lateral Sclerosis (ALS) [94,101,102].

This review will explore the evidence for a role for RIP kinase-regulated cell death and inflammation in rare autoinflammatory syndromes and how mechanistic insights from these conditions can be extrapolated into chronic inflammatory diseases of the gastrointestinal tract, joints, and skin (Table 1), which are triggered by ill-defined environmental and genetic factors. It will also address the therapeutic potential of targeting RIPK1 in the clinic to treat these disorders.

## 2. Multi-Organ—Autoinflammatory Syndromes

To date, mutations in cell death regulatory genes, including TNFR1, XIAP, A20, OTULIN, LUBAC components (HOIL-1, HOIP and SHARPIN), RIPK1, and caspase-8, cause primary immunodeficiency, autoimmunity, and/or trigger autoinflammation reminiscent of NLRP3 autoactivating mutation-associated cryopyrin-associated periodic syndromes (CAPS). In the case of TNFR-associated periodic syndrome (TRAPs), XIAP deficiency (i.e., hemophagocytic lymphohistiocytosis), haploinsufficiency in A20 (HA20), LUBAC deficiency, otulipenia, and biallelic RIPK1 deficiency common overlapping clinical manifestations include recurrent fever and infection, arthritis and arthralgia/myalgia (i.e., joint and musculoskeletal pain), skin lesions, and intestinal inflammation and/or diarrhoea, as well as on occasion hepatosplenomegaly [63,98,103,104,105,106,107,108]. Often, mutation of these genes in mice causes embryonic or perinatal lethality associated with multi-organ inflammation and/or excessive cell death that precludes analysis of common disease manifestations, but cell-specific gene targeting is beginning to establish disease mechanisms and therapeutic targets [107,109,110,111,112,113]. Moreover, despite the variability in death modalities that may drive autoinflammation, therapeutics targeting TNF, IL-6, or IL-1β itself are comparatively effective, although responses can be unpredictable [114]. Hence, targeting upstream signalling pathways may yield a more uniform therapeutic responsiveness and this approach will be discussed in more detail below.

## 3. Joints—Rheumatoid Arthritis

Historically, RA development and chronicity have been linked to a lack of apoptosis due to the expression of intrinsic and extrinsic pro-survival proteins in inflamed synovial joints [115,116,117,118,119]. This prompted interest in examining the cell-specific survival requirements of pathogenic cells [120,121,122,123] and the repurposing and pre-clinical testing of anti-cancer small-molecule drugs (e.g., BH3 mimetics, pegylated TRAIL) to treat arthritis, albeit with limited to moderate therapeutic success [124,125,126,127]. In fact, the generation of conditional mutant mice lacking key extrinsic cell death regulatory machinery, namely A20 (*A20*^LysM.cre^), cIAP1/2 (*cIap1*^LysM.cre^*cIap2*^−/−^), or XIAP/cIAP1/2 (*cIap1*^LysM.cre^*Xiap*^−/−^*cIap2*^−/−^) in myeloid cells, and caspase-8 inhibitor c-FLIP (*c-flar*^CD11c.cre^) in dendritic cells, revealed how perturbation of cell death signalling pathways can induce multi-organ inflammation characterised by severe inflammatory arthritis (Table 1). Moreover, a dominant inflammasome-associated IL-1β signature was observed in all arthritic mutant mice; with the exception of the myeloid-specific cIAP1/2 knockout mouse (that exhibited TNF as the main biomarker) [45,82,128,129]. Notably, pathogenicity of disease in these spontaneous arthritis models is complex as, in some cases, disease is rescued by NLRP3 inflammasome deficiency rather than TNF loss, and vice versa. Therefore, the contribution of apoptotic versus necroptotic signalling, particularly in IL-1β activation, remains ill defined. For example, while in vitro evidence suggests that NLRP3 inflammasome and IL-1β activation in the absence of A20 (encoded by *TNFAIP3*) is regulated by RIPK3-caspase-8 signalling, a recent study in *A20*^LysM.cre^ mice showed that loss of RIPK3, MLKL, or the kinase activity of RIPK1, dampened IL-1β activity and alleviated arthritis symptoms [130].

Examination of genetic knockout mice lacking central cell death repressors, the IAPs, in experimental arthritis models has also revealed potential overlap between monogenic disease and inflammatory arthritis pathogenesis. Reconstitution of wild-type mice with bone marrow lacking XIAP/cIAP1/2 or cIAP1/2 in the myeloid compartment not only transferred a mild form of spontaneous arthritis but also promoted exaggerated innate immune cell-driven K/BxN serum-induced arthritic responses that were associated with enhanced IL-1β and TNF activity, respectively. Noting that single cIAP1-, cIAP2-, and XIAP-deficient, as well as XIAP/cIAP2 doubly-deficient mice behaved akin to wild-type animals [45]. Further mechanistic insight into how TNFR1 and TLR signalling may regulate inflammatory arthritis has also been gained using mice deficient in core cell death signalling machinery and kinase inhibitors. For example, in the acute K/BxN serum-induced arthritis model, signalling via TLR4-MyD88, as well as IL-1α and IL-1β activity, play a more crucial role in disease progression and chronicity than TNF itself [34]. Remarkably, studies examining *Trif*^−/−^, *Ripk3*^−/−^, *Mlkl*^−/−^, and *Ripk3*^−/−^*caspase-8*^−/−^ (where caspase-8 is deleted on a RIPK3-deficient background to avoid lethal necroptotic signalling [43,44]) mice revealed that a TLR-TRIF-RIPK3-caspase-8 signalling axis regulates caspase-1-independent IL-1β secretion during the late macrophage-driven phase of this disease model. Surprisingly, MLKL was redundant for arthritis development or resolution in this context [45]. Fitting with the idea that dysregulated caspase-8 activity may drive disease progression, mice lacking the extrinsic death receptor Fas in myeloid cells also exhibited enhanced disease resolution, although this was not associated with a blunted IL-1β response [123]. Intriguingly, a further study showed that conditional deletion of caspase-8 in dendritic cells (*Caspase-8*^CD11c.cre/+^) exacerbated arthritis, whilst, conversely, myeloid cell-specific caspase-8 loss (*Caspase-8*^LysM.cre/cre^) enhanced disease resolution. Disease perturbations were suggested to be caused by altered RIPK3-mediated signalling and the development of innate immune cell populations, as co-deletion of RIPK3 reversed overall disease responses [131]. This work points to RIPK1/3-caspase-8 signalling having differential effects in specific myeloid populations, as previously evidenced by the distinct role for RIPK1 versus RIPK3 in repressing cytokine production in the *Caspase-8*^CD11c.cre/+^ and *Caspase-8*^LysM.cre^ mice [132]. It is also worth noting that in our hands, *Caspase-8*^LysM.cre/+^ (hemizygous Cre) mice developed comparable, if not mildly enhanced, levels of arthritis compared to *Caspase-8*^lox/lox^ mice (Lawlor KE, unpublished data), which is more reminiscent of the *Caspase-8*^CD11c.cre/+^ mice. Overall, differences in RIPK-caspase-8 signalling requirements in arthritis pathogenesis could be due to differential cell-specific effects, levels of Cre recombinase expression, absolute levels of caspase-8 that can act as scaffolding for multiple modes of cell death [45,69,70,87,132,133], as well as environmental and genetic differences (e.g., *Caspase-8*^lox/lox^ derived on a 129 [131] versus C57BL/6 [45] background).

RIPK1 kinase inhibitors are an attractive therapeutic avenue for RA, as they may limit TNF-induced inflammation in addition to blocking cell death that also has the propensity to be pro-inflammatory. Indeed, two groups have documented the therapeutic efficacy of RIPK1 kinase inhibitors in acute and chronic arthritis models. In the case of chronic autoimmune collagen-induced arthritis, prophylactic treatment with the RIPK1 kinas inhibitor necrostatin-1s (Nec-1s) modestly reduced the incidence and severity of arthritis and was associated with reduced cytokine and necroptotic activity in the synovium, a skewing of the CD4^+^ T-cell response towards a T_H_2 and regulatory T-cell profile, and attenuated osteoclastogenesis [134]. In comparison, both RIPK1 kinase dead mice and mice receiving the RIPK1 kinase inhibitor, GNE684, were shown to have reduced acute innate immune cell-driven collagen antibody-induced arthritis (CAIA). This effect was directly attributable to GNE684 inhibiting TNF signalling, as blocking TNF using TNFR2-Fc caused an equivalent effect and failed to further dampen responses upon co-therapy [135]. On the surface, these results suggest that RIPK1 kinase inhibitors are likely to be as efficacious as TNF biologicals for the treatment of RA. However, a Phase II clinical trial in patients with moderate to severe RA using the first-in-class small-molecule RIPK1 inhibitor GSK2982772 (NCT02858492) failed to see significant clinical improvement beyond potential reductions in bone erosions [136]. It remains to be seen whether improved bioavailability, or treatment of mild RA cases, will reveal therapeutic efficacy.

## 4. Skin—Psoriasis and Dermatitis

RIPK1 has an established role in preventing apoptosis and necroptosis of murine epithelial cells, and *Ripk1*^−/−^ neonates have elevated levels of inflammatory cytokines in their skin, as well as keratinocyte hyperplasia that is a hallmark of excessive cell death [30,35,36,59,61,137]. RIPK1 also appears to play a protective role in human keratinocytes, albeit less dominantly. Patients with biallelic mutations in RIPK1 can present with inflammatory skin lesions among a number of more debilitating pathologies (Table 1) [98,99]. In contrast, patients with cleavage-resistant RIPK1 autoinflammatory (CRIA) syndrome caused by heterozygous mutations in the caspase-8 cleavage site (D324) do not display a skin phenotype, although mice harbouring a homozygous mutation (*Ripk1*^D325A/D325A^) of this conserved site do exhibit skin hyperplasia [138]. In fact, a number of mouse models with dysregulated complex I signalling present with lethal inflammatory skin disorders, often linked to RIPK1 activity [139,140]. In humans, autoinflammatory skin disorders, such as psoriasis, are multi-factorial [141]. However, inflammation-associated keratinocyte cell death does appear to major contributor to disease pathology.

The heterotrimeric linear ubiquitin chain assembly complex (LUBAC; composed of HOIP that harbours the essential enzymatic activity, and HOIL-1 and SHARPIN that promote complex assembly and stability) coordinates NF-κB activation via linear ubiquitylation of RIPK1 and NEMO within complex I. As briefly touched on above, HOIL-1- and HOIP-deficient patients survive but present with immunodeficiency and multi-organ autoinflammation, including dermatitis [106,142,143]. In contrast, the absence of HOIL-1 or HOIP in mice, and the ensuing lack of linear ubiquitylation in complex I, results in early-mid-gestational lethality due to aberrant TNFR1-mediated endothelial apoptotic and necroptotic cell death that is only partially dependent on the kinase activity of RIPK1 [144]. Intriguingly, whilst combined MLKL and caspase-8 deficiency rescues the HOIL-1 and HOIP-deficient mice from embryonic lethality, co-deletion of RIPK3 and caspase-8 results in late-gestation lethality due to RIPK1-induced haematopoietic defects [144]. Examination of the role of LUBAC selectively in skin homeostasis, via conditional deletion of HOIL-1 and HOIP in mouse keratinocytes (keratin 14 promoter), revealed the onset of lethal postnatal dermatitis at 4–6 days-of-age, which was partially attributable to exaggerated TNFR1-mediated apoptotic caspase-8 activity but largely RIPK1-independent [145]. Interestingly, however, the skin lesions that developed in the absence of both TNFR1 and HOIL-1 were delayed by MLKL loss and completely rescued by RIPK1 kinase inhibition using GSK’547, suggesting a combination of RIPK1 kinase-dependent apoptosis and necroptosis is triggered in the absence of LUBAC and TNFR1 [145]. Additionally, in the absence of TNFR1, dermatitis was induced redundantly by TNF-related apoptosis-inducing ligand (TRAIL) or Fas (CD95), suggesting that multiple death receptors mediate skin disease in the absence of linear ubiquitylation signals [145]. 

In contrast to HOIL-1- and HOIP-deficiency, mice deficient in SHARPIN (*Sharpin*^cpdm/cpdm^) are viable but present with severe multi-organ inflammation and progressive dermatitis with alopecia that develops from 3–4 weeks of age [146]. Viability is attributed to the fact that, in the absence of SHARPIN, decoration of TNFR1 signalling components with linear ubiquitin chains is merely reduced, rather than absent [144]. Dermatitis in *Sharpin*^cpdm/cpdm^ mice is driven by TNF/TNFR signalling in keratinocytes and is dependent on the kinase activity of RIPK1, and also partly dependent on NLRP3 inflammasome-mediated IL-1β activity and IL-1 receptor signalling [19,113,140,147,148,149,150]. Caspase-8 heterozygosity significantly delays dermatitis onset, while co-deletion of FADD (in the skin) and RIPK3 was required to entirely ablate skin inflammation, implying that inflammation is primarily driven by apoptosis, with a minor role for necroptosis [113,151]. Interestingly, in line with the late-gestation lethality observed in HOIL-1- and HOIP-deficient mice, *Sharpin*^cpdm/cpdm^*Ripk3*^−/−^*caspase-8*^−/−^ mice exhibited perinatal lethality due to defects in haematopoiesis [113,144]. Mirroring genetic studies, small-molecule RIPK1 inhibitor GNE684 therapy in *Sharpin*^cpdm/cpdm^ mice with established dermatitis reduced inflammation and caspase-3 cleavage in the skin [135,152].

As discussed above, the inhibitor of apoptosis proteins (cIAP1, cIAP2, and XIAP) also regulate cell fate in a RIPK1 and/or RIPK3-dependent manner. Loss of IAP activity in the skin, via injection of a pan-IAP-targeting Smac mimetic (SM), Compound A or 911, leads to more extensive skin lesions than those observed in *Sharpin*^cpdm/cpdm^ mice. Noting that disease manifestations were linked to TNFR1 and/or FasL-mediated apoptotic cell death and inflammation, with necroptosis appearing to drive secondary damage [139]. Genetic loss of specific IAP family members was also found to trigger distinct skin phenotypes. For example, while *cIap1*^K14.cre/K14.cre^*Xiap*^−/−^ mice develop a spongiotic dermatitis with psoriasis-like features affecting the ears and face from around 10 weeks of age, *cIap1*^K14.cre/K14.cre^*cIap2^−/−^* mice become moribund at postpartum day 10 due to widespread dermatoses that, like the *Sharpin*^cpdm/cpdm^ mice, are prevented by *Ripk1* heterozygosity [139]. In humans, IAPs are abundantly expressed in the skin and their levels are potently reduced by SM administration [153], so one could envisage unwanted inflammation could present during systemic therapies. However, SM have in general been well tolerated during Phase1/II clinical trials for haematological and solid cancer malignancies; with only high doses inducing adverse events (e.g., cytokine release syndrome, diarrhoea, vomiting, fatigue, anorexia, and occasionally a pruritic rash [93,154]).

Neutrophilic dermatoses, such as Sweet’s syndrome and pyoderma gangrenosum, are often associated with mutant forms of protein tyrosine phosphatase-6 (PTPN6) that trigger cell death-induced tissue inflammation [155,156]. In mice, conditional deletion of *Ptpn6* in neutrophils is sufficient to initiate a severe IL-1α/β-mediated cutaneous inflammatory disease driven by both caspase-8-dependent apoptotic and RIPK3/MLKL-dependent necroptotic signalling, suggesting that mutant forms of PTPN6 may fail to restrict RIPK1 pro-death activity [157,158]. Remarkably, genetic loss of RIPK1, or its kinase activity (*Ripk1*^D138N^), actually accelerated disease onset in this context, suggesting divergent signalling in neutrophilic dermatoses compared to psoriasiform conditions caused by LUBAC and IAP mutations [158].

Despite genetic evidence that the IAPs and RIPK1 can repress inflammatory skin lesions, studies into RIPK1 and RIPK3 function in the context of psoriasis are limited. RIPK1 expression was reported to be decreased in lesions of plaque-type psoriasis patients, thereby sensitizing keratinocytes to TRAIL. Correspondingly, TRAIL neutralisation in an imiquimod (IMQ)-induced murine model of psoriasis improved skin inflammation and led to a decrease in inflammatory cytokines [159]. In contrast, a more recent study observed increased levels of RIPK1, RIPK3, MLKL, and phosphorylated (p)MLKL in psoriatic lesions from patients, suggesting that the skin pathology may be driven by necroptosis [160]. Consistent with this notion, treatment with the RIPK1 inhibitor, Nec-1s, or the MLKL inhibitor necrosulfonamide (NSA) reduced IMQ-induced psoriasis, with the caveats that NSA targets GSDMD rather than MLKL in mice, and the fact that a further study found no role genetically for the kinase activity of RIPK1 or RIPK3 [161,162]. Overall, the complexity of RIPK1’s role in skin inflammation suggests that caution should be taken when targeting RIPK1 in all hyperinflammatory disorders, or that rationally designed drugs targeting other cell death machinery may have merit [158]. Encouragingly, a recent Phase II clinical trial using RIPK1 inhibitor GSK2982772 in plaque-type psoriasis has shown therapeutic benefit but there is still a lack of data surrounding RIPK1 inhibitors and their long-term use in the clinic [163,164].

## 5. Gut—Inflammatory Bowel Disease

Inflammatory bowel disease (IBD), which includes the chronic relapsing inflammatory disorders Crohn’s Disease (CD) and ulcerative colitis (UC), is characterised by intestinal inflammation and epithelial cell injury affecting the gastrointestinal (GI) lining [165]. The GI tract harbours an enormous bacterial load and disruption of mucosal barrier integrity due to aberrant intestinal epithelial cell (IEC) death allows luminal bacteria to enter the lamina propria and trigger inflammation [166,167,168].

Germline loss-of-function mutations in XIAP pre-dispose males to very-early-onset (VEO)-IBD [169,170,171]. Additionally, approximately 50–70% of patients with X-linked lymphoproliferative disease 2 (XLP2), caused by mutations that limit XIAP expression/function, develop hemophagocytic lymphohistiocytosis (HLH) with symptoms that include hyperinflammation and early-onset chronic haemorrhagic colitis [172,173,174,175]. In addition to its critical role in suppressing ripoptosome formation and subsequent NLRP3 inflammasome activation in innate immune cells [45,46,47,48,49,78], XIAP also ubiquitylates the nucleotide-binding oligomerisation domain (NOD)-2 adaptor, RIPK2 [176]. NOD2 responds to the bacterial cell wall component, muramyl dipeptide (MDP), and signalling via this pathway has been implicated in IBD pathogenesis [177,178,179]. The fact that both XLP2 and very early-onset (VEO)-IBD *XIAP* variants confer increased sensitivity to cell death [180] suggests the possibility of a combined role for perturbed NOD2-RIPK2 signalling and RIPK1-mediated cell death in IBD pathogenesis.

TNFR1 signalling is unequivocally connected with IBD pathogenesis, as best demonstrated by the wide-spread use of anti-TNF therapeutics in IBD patients [181,182], and the clinical benefits observed in pre-clinical IBD models using drugs targeting downstream TLR/TNFR1 inflammatory signalling molecules [183]. Interestingly, despite a clear role for TNFR1 complex I signalling, evidence now suggests that TNFR1-mediated cell death also contributes to IBD (Table 1). Mutations in mice that derepress RIPK1-dependent apoptotic and/or necroptotic IEC death elicit severe chronic intestinal inflammation, akin to that seen in IBD in humans. For example, loss of IKK complex component NEMO in IECs (*Villin* cre) results in spontaneous colitis mediated by TNFR1 signalling and RIPK1 kinase-dependent cell death [135,184,185], while IKKβ protects against colitis induced by *Clostridium difficile* or dextran sodium sulphate (DSS) [186,187]. Loss of upstream TAK1 in IECs triggers TNFR1-dependent (presumably RIPK1-dependent) apoptosis and intestinal inflammation, although colitis and ileitis ultimately develop in the absence of TNFR1 [188]. Whilst deficiencies in IKK or NF-κB have not yet been reported in human IBD patients, patients with ecto-dermal dysplasia with immunodeficiency (EDA-ID) caused by hypomorphic NEMO mutations often suffer from colitis [189,190,191,192]. In addition, *TNFAIP3*, which encodes A20, is a susceptibility locus for CD and UC in humans [193]. Exactly how A20 expression contributes to IBD development is still unclear but, in contrast to other spontaneous diseases caused by conditional A20 loss, deletion of A20 in both IECs and myeloid cells is required to induce spontaneous colitis and ileitis, as well as apoptosis of crypt cells [194,195]. Different groups have also reported that overexpression of A20 in IECs is protective in lipopolysaccharide (LPS) and DSS-induced colitis models [196,197] but promotes RIPK1-dependent IEC death in response to TNF [198].

Both apoptotic caspase-8 and its adaptor protein FADD have been revealed to be vital to maintaining intestinal homeostasis [43,44,199,200]. Mice lacking FADD in IECs (*Fadd*^Villin.cre^) develop severe colitis and ileitis, as well as Paneth cell loss [201]. *Caspase-8^Villin.cre^* mice also present with ileitis and Paneth cell loss, but colitis only develops in the presence of certain microbiota [202,203]. Colitis in both the *Fadd*^Villin.cre^ and *Caspase-8*^Villin.cr*e*^ mice is caused by RIPK1 kinase activity-dependent IEC necroptosis and is primarily mediated by TNFR1, with a minor role for ZBP1 [201,204,205]. In contrast, TNFR1 and ZBP1 act redundantly to trigger ileitis in the *Caspase-8*^Villin.cre^ and *Fadd*^Villin.cre^ mice, which is also driven by necroptotic IEC death. It is worth noting that FADD-deficient IECs can also reportedly utilise RIPK3 scaffolding to undergo caspase-8-mediated apoptosis and a ‘pyroptotic-like’ death [204]. Surprisingly, humans with loss-of-function mutations in the *CASP8* gene develop VEO-IBD that is refractory to TNF treatment [206], suggesting that ZBP1 (or possibly TLR signalling) may also drive intestinal inflammation in human patients. The importance of RIPK1 kinase activity in inflammation arising from loss FADD or caspase-8 loss underscores the therapeutic potential of targeting the kinase activity of RIPK1 in *CASP8* mutant patients with VEO-IBD.

RIPK1 has a critical pro-survival scaffolding role and patients with biallelic mutations in RIPK1 present with early-onset IBD involving the upper and lower GI tract that is triggered by environmental insults [98,99,207]. It is clear from these studies that RIPK1-deficient human cells have aberrant TNFR and TLR signalling responses and a potential increase in the number of apoptotic bodies within crypts. However, how RIPK1 regulates intestinal homeostasis (i.e., IEC death versus cytokine production) remains ill defined. Interestingly, the presence of necrotic lesions in CD patients [202,208], combined with the fact that elevated pRIPK1, pRIPK3, and pMLKL levels in the terminal ileum and colon correlate with severe disease in both CD and UC patients [209,210,211,212], indicates that RIPK1-dependent necroptosis may contribute to pathology in human IBD. Indeed, pharmacological inhibition of RIPK1 kinase activity has been shown to be effective in the murine CD4^+^CD45RB^HI^ T-cell transfer model of colitis and provided dose-dependent protection from inflammatory cell death in *Nemo*^Villin.cre/+^ mice [213]. Yet, curiously, despite some groups reporting that MLKL-deficient mice are protected from DSS-induced colitis and colitis-associated tumourigenesis [214,215], other studies, including those using littermate control animals, found little-to-no role for RIPK1 kinase, RIPK3- or MLKL-dependent necroptotic signalling in DSS-induced colitis and/or colorectal cancer [162,216]. Nonetheless, RIPK1 may still mediate deleterious non-necroptotic signalling in IBD and other related disorders. In fact, pre-clinical studies have shown that the small-molecule RIPK1 kinase inhibitor, GSK2982772, blocks spontaneous cytokine production in explants derived from UC patients and reduces inflammation in a DSS-induced colitis model in mice [164,217]. It will be interesting to see the published results from a recently completed Phase II clinical trial examining the efficacy of GSK2982772 in UC patients [NCT02903966].

## 6. Conclusions

This review gives an overview of the role of RIPK1 and RIPK3 activity in inflammatory diseases associated with the skin, gut, lung, and joints. RIPK1 and RIPK3 are central regulators of inflammatory signalling and control multiple modalities of cell death downstream of death receptor and TLR ligation. As such, perturbations in RIPK-dependent signalling networks are a feature of many auto-inflammatory and chronic inflammatory disorders. Existing therapeutics largely focus on targeting pro-inflammatory cytokines, such as TNF, IL-1β, and IL-6. However, many patients experience treatment failures or severe adverse reactions, supporting the need for new drug development in this space. Small-molecule RIP kinase inhibitors are now emerging in the clinic. Although a large number of pre-clinical studies have shown that targeting RIPK1 kinase activity limits deleterious inflammation in models of acute and chronic inflammation [101], early results from Phase I/II clinical trials with RIPK1 kinase inhibitors have revealed that, although well tolerated, they may not hold up to their full therapeutic promise [136]. As such, much more information is required in order to fully understand the multi-faceted roles these kinases play in regulating cell fate and innate immunity during homeostasis, as well as during infectious and inflammatory disease in humans. Ultimately, this knowledge will enable better rational drug design and the development of co-therapeutic strategies that maximise clinical benefit whilst ameliorating the risk of treatment failures and unwanted side-effects.

## Figures and Tables

**Table 1:** Spontaneous tissue pathologies linked to RIPK signalling.

Gene Name	Mutation	RIP Kinase Signalling	Joint	Skin	Gut	Ref.
*Tnfaip3* **Mouse**	Myeloid-specific A20 knockout (*Tnfaip3*^LysM.cre^)	RIPK1 kinase/RIPK3/MLKL- dependent necroptosis and NLRP3 inflammasome activation	Arthritis			[82,128,130]
	Intestinal epithelium- and myeloid-specific A20 knockout ^B^ (*Tnfaip3*^Villin.cre^*Tnfaip3*^LysM.cre^)	⇑ TNF-induced IEC apoptosis ^C^			Severe colitis	[194,195]
*cIAP1,* *cIAP2* **Mouse**	Myeloid-specific cIAP1 and global knockout of cIAP2 ^A,B^ (*cIap1*^LysM.cre^*cIap*2^−/−^)	⇑ TNF serum levels	Arthritis			[45]
	Epidermal-specific cIAP1 and global knockout of cIAP2 (*cIap1*^E-KO^*cIap2*^−/−^)	TNF-mediated RIPK1- dependent apoptosis		Severe dermatitis		[139]
*cIAP1,* *XIAP* **Mouse**	Epidermal-specific cIAP1 and global knockout of XIAP (*cIap1*^E-KO^ *Xiap*^−/−^)	RIPK1-dependent cell death		Severe dermatitis		[139]
*cIAP1, cIAP2,* *XIAP* **Mouse**	Myeloid-specific cIAP1 and global knockout of cIAP2 and XIAP ^B^ (*cIap1*^LysM.cre^*cIap2*^−/−^ *Xiap*^−/−^)	⇑ TNF, IL-1 RIPK3-dependent apoptosis or necroptosis	Arthritis	Inflammation		[45]
*Fadd* **Mouse**	Intestinal epithelial cell-specific knockout of FADD (*Fadd*^Villin.cre^)	TNF-induced RIPK3- dependent necroptosis Caspase-8/GSDMD- dependent pyroptosis			Colitis, ileitis	[201,204]
*Caspase-8* **Mouse**	IEC-specific knockout of Caspase 8 (*Caspase-8*^Villin.cre^)	TNF-induced RIPK3- dependent necroptosis			Ileitis, susceptible to colitis	[202,203]
**Human**	Loss of function mutations in Caspase-8	⇑ IL-1 (patient-derived monocytes) ^C^			VEO-IBD	[206]
*RIPK1* **Mouse**	Global RIPK1 knockout ^A,B^ (*Ripk1*^−/−^)	Apoptosis and RIPK3- dependent necroptosis		Epidermal hyperplasia		[35,97]
	Epithelial-specific knockout of RIPK1 (*Ripk1*^E-KO^)	TNFR1-induced RIPK3- and ZBP1-RIPK3-MLKL- dependent necroptosis		Psoriasis-like disease		[30,61,137]
	RHIM domain mutation ^A^ (*Ripk1*^RHIM/RHIM^)	Overactive ZBP1-RIPK3-MLKL- dependent necroptosis		Dermatitis		[59]
	IEC-specific knockout of RIPK1 ^A^ (*Ripk1*^Villin.cre^)	TNF/FADD-dependent apoptosis or RIPK3- dependent necroptosis (when FADD is absent)			Inflammation	[30,36]
	Caspase-8 cleavage-resistant RIPK1 ^B^ (*Ripk1*^D325A/D325A^)	TNF-induced apoptosis and necroptosis		Skin hyperplasia		[138]
**Human**	Homozygous loss-of-function or missense mutations ^B^	⇑ IL-1β signalling Aberrant TNFR and TLR signalling	Early- onset poly- arthritis	Skin lesions	IBD	[98,99,138,207]
*HOIL-1* **Mouse**	Keratinocyte-specific deletion of HOIL^A^ (*Hoil*^E-KO^)	TNFR1-induced Caspase-8-dependent apoptosis (RIPK1 independent)		Dermatitis		[145]
**Human**	Autosomal recessive loss-of-function ^B^	⇓ RIPK1 polyubiquitination		Dermatitis	IBD	[143]
*Hoip* **Mouse**	Keratinocyte-specific deletion of HOIP ^A^ (*Hoip*^E-KO^)	TNFR1-induced Caspase-8-dependent RIPK-independent apoptosis		Lethal dermatitis		[145,112]
*Sharpin* **Mouse**	Global knockout (*Sharpin*^cpdm/cpdm^) ^B^	TNFR1-induced cell death ⇓ NF-κB signalling	Arthritis	Severe dermatitis	Loss of Peyer’s patches in gut	[19,113,135,140,147]

Genetic models of spontaneous disease in the joints, skin, or gastrointestinal tract where loss of factors targeting RIPK1/3 directly have been clearly implicated. Key: ^A^ Embryonic or postnatal lethality; ^B^ multi-organ inflammation; ^C^ direct role for RIP kinase signalling is unclear.

## Abbreviations

**A**SC	apoptosis-associated speck-like containing a CARD
**C**AIA	collagen antibody-induced arthritis
**C**APS	cryopyrin-associated periodic syndromes
**C**ARD	caspase activation and recruitment domain
**c**aspase	cysteine-dependent aspartic acid-specific protease
**C**D	Crohn’s Disease
**c**FLIP	cellular FLICE-like inhibitory protein
**c**IAP	cellular inhibitor of apoptosis protein
**C**RIA	cleavage-resistant RIPK1-induced autoinflammatory syndrome
**C**YLD	cylindromatosis
**D**AI	DNA-dependent activator of IFN regulatory transcription factors
**D**AMP	damage-associated molecular pattern
**D**NA	deoxyribonucleic acid
**E**DA-ID	ecto-dermal dysplasia with immunodeficiency
**E**R	endoplasmic reticulum
**G**I	gastrointestinal
**G**SDMD	gasdermin D
**H**A20	haploinsufficiency in A20
**H**LH	hemophagocytic lymphohistiocytosis
**H**MGB1	high-mobility group box 1
**H**OIL-1	haeme-oxidized IRP2 ubiquitin ligase 1
**H**OIP	HOIL1-interacting protein
**H**SP	heat shock protein
**I**BD	inflammatory bowel disease
**I**AV	Influenza A virus
**I**EC	intestinal epithelial cell
**I**FN	interferon
**I**L	interleukin
**I**MQ	imiquimod
**L**OF	loss of function
**L**RR	leucine-rich repeat
**L**UBAC	linear ubiquitin chain assembly complex
**M**APK	mitogen-activated protein kinase
**M**DP	muramyl dipeptide
**M**LKL	mixed lineage kinase domain-like
**N**EMO	NF-κB essential modulator (IKK)
**N**F-κB	nuclear factor-kappa B
**N**LR	NOD-like receptor
**N**LRP3	NOD-like protein receptor 3
**N**OD	nucleotide-binding oligomerization domain
**N**SA	necrosulfonamide
**P**AMP	pathogen-associated molecular pattern
**P**RR	pattern recognition receptor
**R**A	rheumatoid arthritis
**R**IPK	receptor-interacting protein kinase
**R**NA	ribonucleic acid
**R**OS	reactive oxygen species
**R**SV	respiratory syncytial virus
**S**HARPIN	SHANK-associated RH domain-interacting protein
**P**TPN6	protein tyrosine phosphatase-6
**S**MAC	second mitochondrial activator of caspases
**S**M	Smac mimetic
**T**AK1	transforming growth factor—activated kinase
**T**LR	Toll-like receptor
**T**NF	tumour necrosis factor
**T**NFR	tumour necrosis factor receptor
**T**RADD	TNFR1-associated death domain protein
**T**RAF2	TNF receptor-associated factor 2
**T**RAIL	TNF-related apoptosis-inducing ligand
**T**RAPS	TNFR-associated periodic syndrome
**T**RIF	TIR-domain-containing adapter-inducing interferon-β
**U**C	ulcerative colitis
**V**EO	very-early onset
**X**IAP	X-linked inhibitor of apoptosis protein
**X**PL2	X-linked lymphoproliferative disease 2
**Z**BP1	Z-DNA binding protein 1

## References

[1] Furman D., Campisi J., Verdin E., Carrera-Bastos P., Targ S., Franceschi C., Ferrucci L., Gilroy D.W., Fasano A., Miller G.W. (2019). Chronic inflammation in the etiology of disease across the life span. Nat. Med..

[2] Humphries F., Yang S., Wang B., Moynagh P.N. (2015). RIP kinases: Key decision makers in cell death and innate immunity. Cell Death Differ..

[3] He S., Wang X. (2018). RIP kinases as modulators of inflammation and immunity. Nat. Immunol..

[4] Eng V.V., Wemyss M.A., Pearson J.S. (2020). The diverse roles of RIP kinases in host-pathogen interactions. Semin. Cell Dev. Biol..

[5] Meylan E., Burns K., Hofmann K., Blancheteau V., Martinon F., Kelliher M., Tschopp J. (2004). RIP1 is an essential mediator of Toll-like receptor 3-induced NF-kappa B activation. Nat. Immunol..

[6] Kelliher M.A., Grimm S., Ishida Y., Kuo F., Stanger B.Z., Leder P. (1998). The Death Domain Kinase RIP Mediates the TNF-Induced NF-kB Signal. Immunity.

[7] Ting A.T., Pimentel-Muinos F.X., Seed B. (1996). RIP mediates tumor necrosis factor receptor 1 activation of NF-kappaB but not Fas/APO-1-initiated apoptosis. EMBO J..

[8] Holler N., Zaru R., Micheau O., Thome M., Attinger A., Valitutti S., Bodmer J.L., Schneider P., Seed B., Tschopp J. (2000). Fas triggers an alternative, caspase-8-independent cell death pathway using the kinase RIP as effector molecule. Nat. Immunol..

[9] Hsu H., Huang J., Shu H.B., Baichwal V., Goeddel D.V. (1996). TNF-dependent recruitment of the protein kinase RIP to the TNF receptor-1 signaling complex. Immunity.

[10] Park Y.H., Jeong M.S., Park H.H., Jang S.B. (2013). Formation of the death domain complex between FADD and RIP1 proteins In Vitro. Biochim. Biophys. Acta.

[11] Stanger B.Z., Leder P., Lee T.H., Kim E., Seed B. (1995). RIP: A novel protein containing a death domain that interacts with Fas/APO-1 (CD95) in yeast and causes cell death. Cell.

[12] Sun X., Yin J., Starovasnik M.A., Fairbrother W.J., Dixit V.M. (2002). Identification of a novel homotypic interaction motif required for the phosphorylation of receptor-interacting protein (RIP) by RIP3. J. Biol. Chem..

[13] Kaiser W.J., Offermann M.K. (2005). Apoptosis induced by the toll-like receptor adaptor TRIF is dependent on its receptor interacting protein homotypic interaction motif. J. Immunol..

[14] Kaiser W.J., Upton J.W., Mocarski E.S. (2008). Receptor-interacting protein homotypic interaction motif-dependent control of NF-kappa B activation via the DNA-dependent activator of IFN regulatory factors. J. Immunol..

[15] Hsu H., Xiong J., Goeddel D.V. (1995). The TNF receptor 1-associated protein TRADD signals cell death and NF-kappa B activation. Cell.

[16] Micheau O., Tschopp J. (2003). Induction of TNF Receptor I-Mediated Apoptosis via Two Sequential Signaling Complexes. Cell.

[17] Shu H.B., Takeuchi M., Goeddel D.V. (1996). The tumor necrosis factor receptor 2 signal transducers TRAF2 and c-IAP1 are components of the tumor necrosis factor receptor 1 signaling complex. Proc. Natl. Acad. Sci. USA.

[18] Vince J.E., Pantaki D., Feltham R., Mace P.D., Cordier S.M., Schmukle A.C., Davidson A.J., Callus B.A., Wong W.W., Gentle I.E. (2009). TRAF2 Must Bind to Cellular Inhibitors of Apoptosis for Tumor Necrosis Factor (TNF) to Efficiently Activate NF-kB and to Prevent TNF-induced Apoptosis. J. Biol. Chem..

[19] Gerlach B., Cordier S.M., Schmukle A.C., Emmerich C.H., Rieser E., Haas T.L., Webb A.I., Rickard J.A., Anderton H., Wong W.W. (2011). Linear ubiquitination prevents inflammation and regulates immune signalling. Nature.

[20] Haas T.L., Emmerich C.H., Gerlach B., Schmukle A.C., Cordier S.M., Rieser E., Feltham R., Vince J., Warnken U., Wenger T. (2009). Recruitment of the linear ubiquitin chain assembly complex stabilizes the TNF-R1 signaling complex and is required for TNF-mediated gene induction. Mol. Cell.

[21] Tokunaga F., Nakagawa T., Nakahara M., Saeki Y., Taniguchi M., Sakata S., Tanaka K., Nakano H., Iwai K. (2011). SHARPIN is a component of the NF-kappaB-activating linear ubiquitin chain assembly complex. Nature.

[22] Liu Z., Chan F.K. (2020). Regulatory mechanisms of RIPK1 in cell death and inflammation. Semin. Cell Dev. Biol..

[23] Simpson D.S., Gabrielyan A., Feltham R. (2020). RIPK1 ubiquitination: Evidence, correlations and the undefined. Semin. Cell Dev. Biol..

[24] Jaco I., Annibaldi A., Lalaoui N., Wilson R., Tenev T., Laurien L., Kim C., Jamal K., Wicky John S., Liccardi G. (2017). MK2 Phosphorylates RIPK1 to Prevent TNF-Induced Cell Death. Mol. Cell.

[25] Wang L., Du F., Wang X. (2008). TNF-alpha induces two distinct caspase-8 activation pathways. Cell.

[26] Hitomi J., Christofferson D.E., Ng A., Yao J., Degterev A., Xavier R.J., Yuan J. (2008). Identification of a molecular signaling network that regulates a cellular necrotic cell death pathway. Cell.

[27] Dynek J.N., Goncharov T., Dueber E.C., Fedorova A.V., Izrael-Tomasevic A., Phu L., Helgason E., Fairbrother W.J., Deshayes K., Kirkpatrick D.S. (2010). c-IAP1 and UbcH5 promote K11-linked polyubiquitination of RIP1 in TNF signalling. EMBO J..

[28] Dondelinger Y., Aguileta M.A., Goossens V., Dubuisson C., Grootjans S., Dejardin E., Vandenabeele P., Bertrand M.J. (2013). RIPK3 contributes to TNFR1-mediated RIPK1 kinase-dependent apoptosis in conditions of cIAP1/2 depletion or TAK1 kinase inhibition. Cell Death Differ..

[29] Dondelinger Y., Jouan-Lanhouet S., Divert T., Theatre E., Bertin J., Gough P.J., Giansanti P., Heck A.J., Dejardin E., Vandenabeele P. (2015). NF-kappaB-Independent Role of IKKalpha/IKKbeta in Preventing RIPK1 Kinase-Dependent Apoptotic and Necroptotic Cell Death during TNF Signaling. Mol. Cell.

[30] Dannappel M., Vlantis K., Kumari S., Polykratis A., Kim C., Wachsmuth L., Eftychi C., Lin J., Corona T., Hermance N. (2014). RIPK1 maintains epithelial homeostasis by inhibiting apoptosis and necroptosis. Nature.

[31] Dillon C.P., Weinlich R., Rodriguez D.A., Cripps J.G., Quarato G., Gurung P., Verbist K.C., Brewer T.L., Llambi F., Gong Y.N. (2014). RIPK1 blocks early postnatal lethality mediated by caspase-8 and RIPK3. Cell.

[32] Kaiser W.J., Daley-Bauer L.P., Thapa R.J., Mandal P., Berger S.B., Huang C., Sundararajan A., Guo H., Roback L., Speck S.H. (2014). RIP1 suppresses innate immune necrotic as well as apoptotic cell death during mammalian parturition. Proc. Natl. Acad. Sci. USA.

[33] Kearney C.J., Cullen S.P., Clancy D., Martin S.J. (2014). RIPK1 can function as an inhibitor rather than an initiator of RIPK3-dependent necroptosis. FEBS J..

[34] Orozco S., Yatim N., Werner M.R., Tran H., Gunja S.Y., Tait S.W., Albert M.L., Green D.R., Oberst A. (2014). RIPK1 both positively and negatively regulates RIPK3 oligomerization and necroptosis. Cell Death Differ..

[35] Rickard J.A., O’Donnell J.A., Evans J.M., Lalaoui N., Poh A.R., Rogers T., Vince J.E., Lawlor K.E., Ninnis R.L., Anderton H. (2014). RIPK1 regulates RIPK3-MLKL-driven systemic inflammation and emergency hematopoiesis. Cell.

[36] Takahashi N., Vereecke L., Bertrand M.J., Duprez L., Berger S.B., Divert T., Goncalves A., Sze M., Gilbert B., Kourula S. (2014). RIPK1 ensures intestinal homeostasis by protecting the epithelium against apoptosis. Nature.

[37] Feoktistova M., Geserick P., Kellert B., Dimitrova D.P., Langlais C., Hupe M., Cain K., MacFarlane M., Hacker G., Leverkus M. (2011). cIAPs block Ripoptosome formation, a RIP1/caspase-8 containing intracellular cell death complex differentially regulated by cFLIP isoforms. Mol. Cell.

[38] Tenev T., Bianchi K., Darding M., Broemer M., Langlais C., Wallberg F., Zachariou A., Lopez J., MacFarlane M., Cain K. (2011). The Ripoptosome, a signaling platform that assembles in response to genotoxic stress and loss of IAPs. Mol. Cell.

[39] Du C., Fang M., Li Y., Li L., Wang X. (2000). Smac, a mitochondrial protein that promotes cytochrome c-dependent caspase activation by eliminating IAP inhibition. Cell.

[40] Verhagen A.M., Ekert P.G., Pakusch M., Silke J., Connolly L.M., Reid G.E., Moritz R.L., Simpson R.J., Vaux D.L. (2000). Identification of DIABLO, a mammalian protein that promotes apoptosis by binding to and antagonizing IAP proteins. Cell.

[41] Mihaly S.R., Ninomiya-Tsuji J., Morioka S. (2014). TAK1 control of cell death. Cell Death Differ..

[42] Feltham R., Vince J.E., Lawlor K.E. (2017). Caspase-8: Not so silently deadly. Clin. Transl. Immunol..

[43] Oberst A., Dillon C.P., Weinlich R., McCormick L.L., Fitzgerald P., Pop C., Hakem R., Salvesen G.S., Green D.R. (2011). Catalytic activity of the caspase-8-FLIP(L) complex inhibits RIPK3-dependent necrosis. Nature.

[44] Kaiser W.J., Upton J.W., Long A.B., Livingston-Rosanoff D., Daley-Bauer L.P., Hakem R., Caspary T., Mocarski E.S. (2011). RIP3 mediates the embryonic lethality of caspase-8-deficient mice. Nature.

[45] Lawlor K.E., Khan N., Mildenhall A., Gerlic M., Croker B.A., D’Cruz A.A., Hall C., Kaur Spall S., Anderton H., Masters S.L. (2015). RIPK3 promotes cell death and NLRP3 inflammasome activation in the absence of MLKL. Nat. Commun..

[46] Yabal M., Muller N., Adler H., Knies N., Gross C.J., Damgaard R.B., Kanegane H., Ringelhan M., Kaufmann T., Heikenwalder M. (2014). XIAP restricts TNF- and RIP3-dependent cell death and inflammasome activation. Cell Rep..

[47] Wicki S., Gurzeler U., Wei-Lynn Wong W., Jost P.J., Bachmann D., Kaufmann T. (2016). Loss of XIAP facilitates switch to TNFalpha-induced necroptosis in mouse neutrophils. Cell Death Dis..

[48] Lawlor K.E., Feltham R., Yabal M., Conos S.A., Chen K.W., Ziehe S., Grass C., Zhan Y., Nguyen T.A., Hall C. (2017). XIAP Loss Triggers RIPK3- and Caspase-8-Driven IL-1beta Activation and Cell Death as a Consequence of TLR-MyD88-Induced cIAP1-TRAF2 Degradation. Cell Rep..

[49] Chen K.W., Lawlor K.E., von Pein J.B., Boucher D., Gerlic M., Croker B.A., Bezbradica J.S., Vince J.E., Schroder K. (2018). Cutting Edge: Blockade of Inhibitor of Apoptosis Proteins Sensitizes Neutrophils to TNF- but Not Lipopolysaccharide-Mediated Cell Death and IL-1beta Secretion. J. Immunol..

[50] He S., Liang Y., Shao F., Wang X. (2011). Toll-like receptors activate programmed necrosis in macrophages through a receptor-interacting kinase-3-mediated pathway. Proc. Natl. Acad. Sci. USA.

[51] He S., Wang L., Miao L., Wang T., Du F., Zhao L., Wang X. (2009). Receptor interacting protein kinase-3 determines cellular necrotic response to TNF-alpha. Cell.

[52] Mompean M., Li W., Li J., Laage S., Siemer A.B., Bozkurt G., Wu H., McDermott A.E. (2018). The Structure of the Necrosome RIPK1-RIPK3 Core, a Human Hetero-Amyloid Signaling Complex. Cell.

[53] Kaczmarek A., Vandenabeele P., Krysko D.V. (2013). Necroptosis: The release of damage-associated molecular patterns and its physiological relevance. Immunity.

[54] Murphy J.M., Czabotar P.E., Hildebrand J.M., Lucet I.S., Zhang J.G., Alvarez-Diaz S., Lewis R., Lalaoui N., Metcalf D., Webb A.I. (2013). The pseudokinase MLKL mediates necroptosis via a molecular switch mechanism. Immunity.

[55] Cai Z., Jitkaew S., Zhao J., Chiang H.C., Choksi S., Liu J., Ward Y., Wu L.G., Liu Z.G. (2014). Plasma membrane translocation of trimerized MLKL protein is required for TNF-induced necroptosis. Nat. Cell Biol..

[56] Dondelinger Y., Declercq W., Montessuit S., Roelandt R., Goncalves A., Bruggeman I., Hulpiau P., Weber K., Sehon C.A., Marquis R.W. (2014). MLKL compromises plasma membrane integrity by binding to phosphatidylinositol phosphates. Cell Rep..

[57] Hildebrand J.M., Tanzer M.C., Lucet I.S., Young S.N., Spall S.K., Sharma P., Pierotti C., Garnier J.M., Dobson R.C., Webb A.I. (2014). Activation of the pseudokinase MLKL unleashes the four-helix bundle domain to induce membrane localization and necroptotic cell death. Proc. Natl. Acad. Sci. USA.

[58] Shlomovitz I., Erlich Z., Speir M., Zargarian S., Baram N., Engler M., Edry-Botzer L., Munitz A., Croker B.A., Gerlic M. (2019). Necroptosis directly induces the release of full-length biologically active IL-33 In Vitro and in an inflammatory disease model. FEBS J..

[59] Newton K., Wickliffe K.E., Maltzman A., Dugger D.L., Strasser A., Pham V.C., Lill J.R., Roose-Girma M., Warming S., Solon M. (2016). RIPK1 inhibits ZBP1-driven necroptosis during development. Nature.

[60] Upton J.W., Kaiser W.J., Mocarski E.S. (2012). DAI/ZBP1/DLM-1 complexes with RIP3 to mediate virus-induced programmed necrosis that is targeted by murine cytomegalovirus vIRA. Cell Host Microbe.

[61] Lin J., Kumari S., Kim C., Van T.M., Wachsmuth L., Polykratis A., Pasparakis M. (2016). RIPK1 counteracts ZBP1-mediated necroptosis to inhibit inflammation. Nature.

[62] Khan N., Lawlor K.E., Murphy J.M., Vince J.E. (2014). More to life than death: Molecular determinants of necroptotic and non-necroptotic RIP3 kinase signaling. Curr. Opin. Immunol..

[63] Speir M., Lawlor K.E. (2020). RIP-roaring inflammation: RIPK1 and RIPK3 driven NLRP3 inflammasome activation and autoinflammatory disease. Semin. Cell Dev. Biol..

[64] Gurung P., Anand P.K., Malireddi R.K., Vande Walle L., Van Opdenbosch N., Dillon C.P., Weinlich R., Green D.R., Lamkanfi M., Kanneganti T.D. (2014). FADD and caspase-8 mediate priming and activation of the canonical and noncanonical Nlrp3 inflammasomes. J. Immunol..

[65] Allam R., Lawlor K.E., Yu E.C., Mildenhall A.L., Moujalled D.M., Lewis R.S., Ke F., Mason K.D., White M.J., Stacey K.J. (2014). Mitochondrial apoptosis is dispensable for NLRP3 inflammasome activation but non-apoptotic caspase-8 is required for inflammasome priming. EMBO Rep..

[66] Weng D., Marty-Roix R., Ganesan S., Proulx M.K., Vladimer G.I., Kaiser W.J., Mocarski E.S., Pouliot K., Chan F.K., Kelliher M.A. (2014). Caspase-8 and RIP kinases regulate bacteria-induced innate immune responses and cell death. Proc. Natl. Acad. Sci. USA.

[67] Zhang X., Fan C., Zhang H., Zhao Q., Liu Y., Xu C., Xie Q., Wu X., Yu X., Zhang J. (2016). MLKL and FADD Are Critical for Suppressing Progressive Lymphoproliferative Disease and Activating the NLRP3 Inflammasome. Cell Rep..

[68] Philip N.H., DeLaney A., Peterson L.W., Santos-Marrero M., Grier J.T., Sun Y., Wynosky-Dolfi M.A., Zwack E.E., Hu B., Olsen T.M. (2016). Activity of Uncleaved Caspase-8 Controls Anti-bacterial Immune Defense and TLR-Induced Cytokine Production Independent of Cell Death. PLoS Pathog..

[69] Kang S., Fernandes-Alnemri T., Rogers C., Mayes L., Wang Y., Dillon C., Roback L., Kaiser W., Oberst A., Sagara J. (2015). Caspase-8 scaffolding function and MLKL regulate NLRP3 inflammasome activation downstream of TLR3. Nat. Commun..

[70] Newton K., Wickliffe K.E., Maltzman A., Dugger D.L., Reja R., Zhang Y., Roose-Girma M., Modrusan Z., Sagolla M.S., Webster J.D. (2019). Activity of caspase-8 determines plasticity between cell death pathways. Nature.

[71] Antonopoulos C., Russo H.M., El Sanadi C., Martin B.N., Li X., Kaiser W.J., Mocarski E.S., Dubyak G.R. (2015). Caspase-8 as an Effector and Regulator of NLRP3 Inflammasome Signaling. J. Biol. Chem..

[72] Sagulenko V., Thygesen S.J., Sester D.P., Idris A., Cridland J.A., Vajjhala P.R., Roberts T.L., Schroder K., Vince J.E., Hill J.M. (2013). AIM2 and NLRP3 inflammasomes activate both apoptotic and pyroptotic death pathways via ASC. Cell Death Differ..

[73] Schneider K.S., Gross C.J., Dreier R.F., Saller B.S., Mishra R., Gorka O., Heilig R., Meunier E., Dick M.S., Cikovic T. (2017). The Inflammasome Drives GSDMD-Independent Secondary Pyroptosis and IL-1 Release in the Absence of Caspase-1 Protease Activity. Cell Rep..

[74] Gaidt M.M., Ebert T.S., Chauhan D., Schmidt T., Schmid-Burgk J.L., Rapino F., Robertson A.A., Cooper M.A., Graf T., Hornung V. (2016). Human Monocytes Engage an Alternative Inflammasome Pathway. Immunity.

[75] Moriwaki K., Bertin J., Gough P.J., Chan F.K. (2015). A RIPK3-caspase 8 complex mediates atypical pro-IL-1beta processing. J. Immunol..

[76] Dondelinger Y., Delanghe T., Priem D., Wynosky-Dolfi M.A., Sorobetea D., Rojas-Rivera D., Giansanti P., Roelandt R., Gropengiesser J., Ruckdeschel K. (2019). Serine 25 phosphorylation inhibits RIPK1 kinase-dependent cell death in models of infection and inflammation. Nat. Commun..

[77] Menon M.B., Gropengiesser J., Fischer J., Novikova L., Deuretzbacher A., Lafera J., Schimmeck H., Czymmeck N., Ronkina N., Kotlyarov A. (2017). p38(MAPK)/MK2-dependent phosphorylation controls cytotoxic RIPK1 signalling in inflammation and infection. Nat. Cell Biol..

[78] Vince J.E., Wong W.W., Gentle I., Lawlor K.E., Allam R., O’Reilly L., Mason K., Gross O., Ma S., Guarda G. (2012). Inhibitor of apoptosis proteins limit RIP3 kinase-dependent interleukin-1 activation. Immunity.

[79] Orning P., Weng D., Starheim K., Ratner D., Best Z., Lee B., Brooks A., Xia S., Wu H., Kelliher M.A. (2018). Pathogen blockade of TAK1 triggers caspase-8-dependent cleavage of gasdermin D and cell death. Science.

[80] Malireddi R.K.S., Gurung P., Mavuluri J., Dasari T.K., Klco J.M., Chi H., Kanneganti T.D. (2018). TAK1 restricts spontaneous NLRP3 activation and cell death to control myeloid proliferation. J. Exp. Med..

[81] Duong B.H., Onizawa M., Oses-Prieto J.A., Advincula R., Burlingame A., Malynn B.A., Ma A. (2015). A20 restricts ubiquitination of pro-interleukin-1beta protein complexes and suppresses NLRP3 inflammasome activity. Immunity.

[82] Vande Walle L., Van Opdenbosch N., Jacques P., Fossoul A., Verheugen E., Vogel P., Beyaert R., Elewaut D., Kanneganti T.D., van Loo G. (2014). Negative regulation of the NLRP3 inflammasome by A20 protects against arthritis. Nature.

[83] Malireddi R.K.S., Gurung P., Kesavardhana S., Samir P., Burton A., Mummareddy H., Vogel P., Pelletier S., Burgula S., Kanneganti T.D. (2020). Innate immune priming in the absence of TAK1 drives RIPK1 kinase activity-independent pyroptosis, apoptosis, necroptosis, and inflammatory disease. J. Exp. Med..

[84] Zheng M., Williams E.P., Malireddi R.K.S., Karki R., Banoth B., Burton A., Webby R., Channappanavar R., Jonsson C.B., Kanneganti T.D. (2020). Impaired NLRP3 inflammasome activation/pyroptosis leads to robust inflammatory cell death via caspase-8/RIPK3 during coronavirus infection. J. Biol. Chem..

[85] Kuriakose T., Man S.M., Malireddi R.K., Karki R., Kesavardhana S., Place D.E., Neale G., Vogel P., Kanneganti T.D. (2016). ZBP1/DAI is an innate sensor of influenza virus triggering the NLRP3 inflammasome and programmed cell death pathways. Sci. Immunol..

[86] Conos S.A., Chen K.W., De Nardo D., Hara H., Whitehead L., Núñez G., Masters S.L., Murphy J.M., Schroder K., Vaux D.L. (2017). Active MLKL triggers the NLRP3 inflammasome in a cell-intrinsic manner. Proc. Natl. Acad. Sci. USA.

[87] Kang T.B., Yang S.H., Toth B., Kovalenko A., Wallach D. (2013). Caspase-8 blocks kinase RIPK3-mediated activation of the NLRP3 inflammasome. Immunity.

[88] Knop J., Spilgies L.M., Rufli S., Reinhart R., Vasilikos L., Yabal M., Owsley E., Jost P.J., Marsh R.A., Wajant H. (2019). TNFR2 induced priming of the inflammasome leads to a RIPK1-dependent cell death in the absence of XIAP. Cell Death Dis..

[89] Gottlieb A.B., Blauvelt A., Thaci D., Leonardi C.L., Poulin Y., Drew J., Peterson L., Arendt C., Burge D., Reich K. (2018). Certolizumab pegol for the treatment of chronic plaque psoriasis: Results through 48 weeks from 2 phase 3, multicenter, randomized, double-blinded, placebo-controlled studies (CIMPASI-1 and CIMPASI-2). J. Am. Acad. Dermatol..

[90] Lebwohl M., Blauvelt A., Paul C., Sofen H., Weglowska J., Piguet V., Burge D., Rolleri R., Drew J., Peterson L. (2018). Certolizumab pegol for the treatment of chronic plaque psoriasis: Results through 48 weeks of a phase 3, multicenter, randomized, double-blind, etanercept- and placebo-controlled study (CIMPACT). J. Am. Acad. Dermatol..

[91] Yamauchi P.S., Bissonnette R., Teixeira H.D., Valdecantos W.C. (2016). Systematic review of efficacy of anti-tumor necrosis factor (TNF) therapy in patients with psoriasis previously treated with a different anti-TNF agent. J. Am. Acad. Dermatol..

[92] Dinarello C.A. (2011). Interleukin-1 in the pathogenesis and treatment of inflammatory diseases. Blood.

[93] Jensen S., Seidelin J.B., LaCasse E.C., Nielson O.H. (2020). SMAC mimetics and RIPK inhibitors as therapeutics for chronic inflammatory diseases. Sci. Signal..

[94] Martens S., Hofmans S., Declercq W., Augustyns K., Vandenabeele P. (2020). Inhibitors Targeting RIPK1/RIPK3: Old and New Drugs. Trends Pharmacol. Sci..

[95] Mandal P., Berger S.B., Pillay S., Moriwaki K., Huang C., Guo H., Lich J.D., Finger J., Kasparcova V., Votta B. (2014). RIP3 induces apoptosis independent of pronecrotic kinase activity. Mol. Cell.

[96] Newton K., Dugger D.L., Wickliffe K.E., Kapoor N. (2014). Activity of protein kinase RIPK3 determines whether cells die by necroptosis or apoptosis. Science.

[97] Polykratis A., Hermance N., Zelic M., Roderick J., Kim C., Van T.M., Lee T.H., Chan F.K.M., Pasparakis M., Kelliher M.A. (2014). Cutting edge: RIPK1 Kinase inactive mice are viable and protected from TNF-induced necroptosis in vivo. J. Immunol..

[98] Cuchet-Lourenco D., Eletto D., Wu C., Plagnol V., Papapietro O., Curtis J., Ceron-Gutierrez L., Bacon C.M., Hackett S., Alsaleem B. (2018). Biallelic RIPK1 mutations in humans cause severe immunodeficiency, arthritis, and intestinal inflammation. Science.

[99] Li Y., Fuhrer M., Bahrami E., Socha P., Klaudel-Dreszler M., Bouzidi A., Liu Y., Lehle A.S., Magg T., Hollizeck S. (2019). Human RIPK1 deficiency causes combined immunodeficiency and inflammatory bowel diseases. Proc. Natl. Acad. Sci. USA.

[100] Degterev A., Hitomi J., Germscheid M., Ch’en I.L., Korkina O., Teng X., Abbott D., Cuny G.D., Yuan C., Wagner G. (2008). Identification of RIP1 kinase as a specific cellular target of necrostatins. Nat. Chem. Biol..

[101] Mifflin L., Ofengeim D., Yuan J. (2020). Receptor-interacting protein kinase 1 (RIPK1) as a therapeutic target. Nat. Rev. Drug Discov..

[102] Weisel K., Scott N.E., Tompson D.J., Votta B.J., Madhavan S., Povey K., Wolstenholme A., Simeoni M., Rudo T., Richards-Peterson L. (2017). Randomized clinical study of safety, pharmacokinetics, and pharmacodynamics of RIPK1 inhibitor GSK2982772 in healthy volunteers. Pharmacol. Res. Perspect..

[103] Aksentijevich I., Galon J., Soares M., Mansfield E., Hull K., Oh H., Goldbach-Mansky R., Dean B., Athreya A., Reginato J. (2001). The tumor-necrosis-factor receptor-associated periodic syndrome: New mutations in TNFRSF1A, ancestral origins, genotype-phenotype studies, and evidence for further genetic heterogeneity of periodic fevers. Am. J. Hum. Genet..

[104] Shabani M., Razaghian A., Alimadadi H., Shiari R., Shahrooei M., Parvaneh N. (2019). Different phenotypes of the same XIAP mutation in a family: A case of XIAP deficiency with juvenile idiopathic arthritis. Pediatr. Blood Cancer.

[105] Zhou Q., Yu X., Demirkaya E., Deuitch N., Stone D., Tsai W.L., Kuehn H.S., Wang H., Yang D., Park Y.H. (2016). Biallelic hypomorphic mutations in a linear deubiquitinase define otulipenia, an early-onset autoinflammatory disease. Proc. Natl. Acad. Sci. USA.

[106] Oda H., Beck D.B., Kuehn H.S., Sampaio Moura N., Hoffmann P., Ibarra M., Stoddard J., Tsai W.L., Gutierrez-Cruz G., Gadina M. (2019). Second Case of HOIP Deficiency Expands Clinical Features and Defines Inflammatory Transcriptome Regulated by LUBAC. Front. Immunol..

[107] Damgaard R.B., Walker J.A., Marco-Casanova P., Morgan N.V., Titheradge H.L., Elliott P.R., McHale D., Maher E.R., McKenzie A.N.J., Komander D. (2016). The Deubiquitinase OTULIN Is an Essential Negative Regulator of Inflammation and Autoimmunity. Cell.

[108] Tsuchida N., Kirino Y., Soejima Y., Onodera M., Arai K., Tamura E., Ishikawa T., Kawai T., Uchiyama T., Nomura S. (2019). Haploinsufficiency of A20 caused by a novel nonsense variant or entire deletion of TNFAIP3 is clinically distinct from Behcet’s disease. Arthritis Res. Ther..

[109] Moulin M., Anderton H., Voss A.K., Thomas T., Wong W.W., Bankovacki A., Feltham R., Chau D., Cook W.D., Silke J. (2012). IAPs limit activation of RIP kinases by TNF receptor 1 during development. EMBO J..

[110] Onizawa M., Oshima S., Schulze-Topphoff U., Oses-Prieto J.A., Lu T., Tavares R., Prodhomme T., Duong B., Whang M.I., Advincula R. (2015). The ubiquitin-modifying enzyme A20 restricts ubiquitination of the kinase RIPK3 and protects cells from necroptosis. Nat. Immunol..

[111] Turer E.E., Tavares R.M., Mortier E., Hitotsumatsu O., Advincula R., Lee B., Shifrin N., Malynn B.A., Ma A. (2008). Homeostatic MyD88-dependent signals cause lethal inflamMation in the absence of A20. J. Exp. Med..

[112] Peltzer N., Rieser E., Taraborrelli L., Draber P., Darding M., Pernaute B., Shimizu Y., Sarr A., Draberova H., Montinaro A. (2014). HOIP deficiency causes embryonic lethality by aberrant TNFR1-mediated endothelial cell death. Cell Rep..

[113] Rickard J.A., Anderton H., Etemadi N., Nachbur U., Darding M., Peltzer N., Lalaoui N., Lawlor K.E., Vanyai H., Hall C. (2014). TNFR1-dependent cell death drives inflammation in Sharpin-deficient mice. Elife.

[114] Ostrov B.E. (2015). Immunotherapeutic Biologic Agents in Autoimmune and Autoinflammatory Diseases. Immunol. Investig..

[115] Matsumoto S., Müller-Ladner U., Gay R.E., Nishioka K., Gay S. (1996). Ultrastructural demonstration of apoptosis, Fas and Bcl-2 expression of rheumatoid synovial fibroblasts. J. Rheumatol..

[116] Liu H., Huang Q., Shi B., Eksarko P., Temkin V., Pope R.M. (2006). Regulation of Mcl-1 expression in rheumatoid arthritis synovial macrophages. Arthritis Rheum..

[117] Dharmapatni A.A., Smith M.D., Findlay D.M., Holding C.A., Evdokiou A., Ahern M.J., Weedon H., Chen P., Screaton G., Xu X.N. (2009). Elevated expression of caspase-3 inhibitors, survivin and xIAP correlates with low levels of apoptosis in active rheumatoid synovium. Arthritis Res. Ther..

[118] Bai S., Liu H., Chen K.H., Eksarko P., Perlman H., Moore T.L., Pope R.M. (2004). NF-kappaB-regulated expression of cellular FLIP protects rheumatoid arthritis synovial fibroblasts from tumor necrosis factor alpha-mediated apoptosis. Arthritis Rheum..

[119] Liu H., Eksarko P., Temkin V., Haines G.K., Perlman H., Koch A.E., Thimmapaya B., Pope R.M. (2005). Mcl-1 is essential for the survival of synovial fibroblasts in rheumatoid arthritis. J. Immunol..

[120] Chen Y., Rosloniec E., Price J., Boothby M., Chen J. (2002). Constitutive expression of BCL-X(L) in the T lineage attenuates collagen-induced arthritis in Bcl-X(L) transgenic mice. Arthritis Rheum..

[121] Zheng B., Marinova E., Switzer K., Wansley D., He H., Bheekha-Escura R., Behrens T.W., Han S. (2007). Overexpression of Bcl(XL) in B cells promotes Th1 response and exacerbates collagen-induced arthritis. J. Immunol..

[122] Lawlor K.E., van Nieuwenhuijze A., Parker K.L., Drake S.F., Campbell I.K., Smith S.D., Vince J.E., Strasser A., Wicks I.P. (2013). Bcl-2 overexpression ameliorates immune complex-mediated arthritis by altering FcgammaRIIb expression and monocyte homeostasis. J. Leukoc. Biol..

[123] Huang Q.Q., Birkett R., Koessler R.E., Cuda C.M., Haines G.K., Jin J.P., Perlman H., Pope R.M. (2014). Fas signaling in macrophages promotes chronicity in K/BxN serum-induced arthritis. Arthritis Rheumatol..

[124] Bardwell P.D., Gu J., McCarthy D., Wallace C., Bryant S., Goess C., Mathieu S., Grinnell C., Erickson J., Rosenberg S.H. (2009). The Bcl-2 family antagonist ABT-737 significantly inhibits multiple animal models of autoimmunity. J. Immunol..

[125] Dharmapatni A.A., Cantley M.D., Marino V., Perilli E., Crotti T.N., Smith M.D., Haynes D.R. (2015). The X-Linked Inhibitor of Apoptosis Protein Inhibitor Embelin Suppresses Inflammation and Bone Erosion in Collagen Antibody Induced Arthritis Mice. Mediat. Inflamm..

[126] Scatizzi J.C., Hutcheson J., Pope R.M., Firestein G.S., Koch A.E., Mavers M., Smason A., Agrawal H., Haines G.K., Chandel N.S. (2010). Bim-Bcl-2 homology 3 mimetic therapy is effective at suppressing inflammatory arthritis through the activation of myeloid cell apoptosis. Arthritis Rheum..

[127] Park J.S., Oh Y., Park O., Foss C.A., Lim S.M., Jo D.G., Na D.H., Pomper M.G., Lee K.C., Lee S. (2017). PEGylated TRAIL ameliorates experimental inflammatory arthritis by regulation of Th17 cells and regulatory T cells. J. Control. Release.

[128] Matmati M., Jacques P., Maelfait J., Verheugen E., Kool M., Sze M., Geboes L., Louagie E., Mc Guire C., Vereecke L. (2011). A20 (TNFAIP3) deficiency in myeloid cells triggers erosive polyarthritis resembling rheumatoid arthritis. Nat. Genet..

[129] Huang Q.Q., Perlman H., Birkett R., Doyle R., Fang D., Haines G.K., Robinson W., Datta S., Huang Z., Li Q.Z. (2015). CD11c-mediated deletion of Flip promotes autoreactivity and inflammatory arthritis. Nat. Commun..

[130] Polykratis A., Martens A., Eren R.O., Shirasaki Y., Yamagishi M., Yamaguchi Y., Uemura S., Miura M., Holzmann B., Kollias G. (2019). A20 prevents inflammasome-dependent arthritis by inhibiting macrophage necroptosis through its ZnF7 ubiquitin-binding domain. Nat. Cell Biol..

[131] Dominguez S., Montgomery A.B., Haines G.K., Bloomfield C.L., Cuda C.M. (2017). The caspase-8/RIPK3 signaling axis in antigen presenting cells controls the inflammatory arthritic response. Arthritis Res. Ther..

[132] Cuda C.M., Misharin A.V., Khare S., Saber R., Tsai F., Archer A.M., Homan P.J., Haines G.K., Hutcheson J., Dorfleutner A. (2015). Conditional deletion of caspase-8 in macrophages alters macrophage activation in a RIPK-dependent manner. Arthritis Res. Ther..

[133] Vince J.E., De Nardo D., Gao W., Vince A.J., Hall C., McArthur K., Simpson D., Vijayaraj S., Lindqvist L.M., Bouillet P. (2018). The Mitochondrial Apoptotic Effectors BAX/BAK Activate Caspase-3 and -7 to Trigger NLRP3 Inflammasome and Caspase-8 Driven IL-1beta Activation. Cell Rep..

[134] Jhun J., Lee S.H., Kim S.Y., Ryu J., Kwon J.Y., Na H.S., Jung K., Moon S.J., Cho M.L., Min J.K. (2019). RIPK1 inhibition attenuates experimental autoimmune arthritis via suppression of osteoclastogenesis. J. Transl. Med..

[135] Patel S., Webster J.D., Varfolomeev E., Kwon Y.C., Cheng J.H., Zhang J., Dugger D.L., Wickliffe K.E., Maltzman A., Sujatha-Bhaskar S. (2019). RIP1 inhibition blocks inflammatory diseases but not tumor growth or metastases. Cell Death Differ..

[136] Weisel K., Berger S., Thorn K., Taylor P.C., Peterfy C., Siddall H., Tompson D., Wang S., Quattrocchi E., Burriss S.W. (2021). A randomized, placebo-controlled experimental medicine study of RIPK1 inhibitor GSK2982772 in patients with moderate to severe rheumatoid arthritis. Arthritis Res. Ther..

[137] Devos M., Tanghe G., Gilbert B., Dierick E., Verheirstraeten M., Nemegeer J., de Reuver R., Lefebvre S., De Munck J., Rehwinkel J. (2020). Sensing of endogenous nucleic acids by ZBP1 induces keratinocyte necroptosis and skin inflammation. J. Exp. Med..

[138] Lalaoui N., Boyden S.E., Oda H., Wood G.M., Stone D.L., Chau D., Liu L., Stoffels M., Kratina T., Lawlor K.E. (2019). Mutations that prevent caspase cleavage of RIPK1 cause autoinflammatory disease. Nature.

[139] Anderton H., Rickard J.A., Varigos G.A., Lalaoui N., Silke J. (2017). Inhibitor of Apoptosis Proteins (IAPs) Limit RIPK1-Mediated Skin Inflammation. J. Investig. Dermatol..

[140] Berger S.B., Kasparcova V., Hoffman S., Swift B., Dare L., Schaeffer M., Capriotti C., Cook M., Finger J., Hughes-Earle A. (2014). Cutting Edge: RIP1 kinase activity is dispensable for normal development but is a key regulator of inflammation in SHARPIN-deficient mice. J. Immunol..

[141] Rendon A., Schakel K. (2019). Psoriasis Pathogenesis and Treatment. Int. J. Mol. Sci..

[142] Boisson B., Laplantine E., Dobbs K., Cobat A., Tarantino N., Hazen M., Lidov H.G., Hopkins G., Du L., Belkadi A. (2015). Human HOIP and LUBAC deficiency underlies autoinflammation, immunodeficiency, amylopectinosis, and lymphangiectasia. J. Exp. Med..

[143] Boisson B., Laplantine E., Prando C., Giliani S., Israelsson E., Xu Z., Abhyankar A., Israel L., Trevejo-Nunez G., Bogunovic D. (2012). Immunodeficiency, autoinflammation and amylopectinosis in humans with inherited HOIL-1 and LUBAC deficiency. Nat. Immunol..

[144] Peltzer N., Darding M., Montinaro A., Draber P., Draberova H., Kupka S., Rieser E., Fisher A., Hutchinson C., Taraborrelli L. (2018). LUBAC is essential for embryogenesis by preventing cell death and enabling haematopoiesis. Nature.

[145] Taraborrelli L., Peltzer N., Montinaro A., Kupka S., Rieser E., Hartwig T., Sarr A., Darding M., Draber P., Haas T.L. (2018). LUBAC prevents lethal dermatitis by inhibiting cell death induced by TNF, TRAIL and CD95L. Nat. Commun..

[146] Seymour R.E., Hasham M.G., Cox G.A., Shultz L.D., Hogenesch H., Roopenian D.C., Sundberg J.P. (2007). Spontaneous mutations in the mouse Sharpin gene result in multiorgan inflammation, immune system dysregulation and dermatitis. Genes Immun..

[147] Ikeda F., Deribe Y.L., Skanland S.S., Stieglitz B., Grabbe C., Franz-Wachtel M., van Wijk S.J., Goswami P., Nagy V., Terzic J. (2011). SHARPIN forms a linear ubiquitin ligase complex regulating NF-kappaB activity and apoptosis. Nature.

[148] Douglas T., Champagne C., Morizot A., Lapointe J.M., Saleh M. (2015). The Inflammatory Caspases-1 and -11 Mediate the Pathogenesis of Dermatitis in Sharpin-Deficient Mice. J. Immunol..

[149] Gurung P., Lamkanfi M., Kanneganti T.D. (2015). Cutting edge: SHARPIN is required for optimal NLRP3 inflammasome activation. J. Immunol..

[150] Gurung P., Sharma B.R., Kanneganti T.D. (2016). Distinct role of IL-1beta in instigating disease in Sharpin(cpdm) mice. Sci. Rep..

[151] Kumari S., Redouane Y., Lopez-Mosqueda J., Shiraishi R., Romanowska M., Lutzmayer S., Kuiper J., Martinez C., Dikic I., Pasparakis M. (2014). Sharpin prevents skin inflammation by inhibiting TNFR1-induced keratinocyte apoptosis. eLife.

[152] Webster J.D., Kwon Y.C., Park S., Zhang H., Corr N., Ljumanovic N., Adedeji A.O., Varfolomeev E., Goncharov T., Preston J. (2020). RIP1 kinase activity is critical for skin inflammation but not for viral propagation. J. Leukoc. Biol..

[153] Infante J.R., Dees E.C., Olszanski A.J., Dhuria S.V., Sen S., Cameron S., Cohen R.B. (2014). Phase I dose-escalation study of LCL161, an oral inhibitor of apoptosis proteins inhibitor, in patients with advanced solid tumors. J. Clin. Oncol..

[154] Morrish E., Brumatti G., Silke J. (2020). Future Therapeutic Directions for Smac-Mimetics. Cells.

[155] Nesterovitch A.B., Gyorfy Z., Hoffman M.D., Moore E.C., Elbuluk N., Tryniszewska B., Rauch T.A., Simon M., Kang S., Fisher G.J. (2011). Alteration in the gene encoding protein tyrosine phosphatase nonreceptor type 6 (PTPN6/SHP1) may contribute to neutrophilic dermatoses. Am. J. Pathol..

[156] Lukens J.R., Vogel P., Johnson G.R., Kelliher M.A., Iwakura Y., Lamkanfi M., Kanneganti T.D. (2013). RIP1-driven autoinflammation targets IL-1alpha independently of inflammasomes and RIP3. Nature.

[157] Abram C.L., Roberge G.L., Pao L.I., Neel B.G., Lowell C.A. (2013). Distinct roles for neutrophils and dendritic cells in inflammation and autoimmunity in motheaten mice. Immunity.

[158] Speir M., Nowell C.J., Chen A.A., O’Donnell J.A., Shamie I.S., Lakin P.R., D’Cruz A.A., Braun R.O., Babon J.J., Lewis R.S. (2019). Ptpn6 inhibits caspase-8- and Ripk3/Mlkl-dependent inflammation. Nat. Immunol..

[159] Saito N., Honma M., Shibuya T., Iinuma S., Igawa S., Kishibe M., Ishida-Yamamoto A. (2018). RIPK1 downregulation in keratinocyte enhances TRAIL signaling in psoriasis. J. Dermatol. Sci..

[160] Duan X., Liu X., Liu N., Huang Y., Jin Z., Zhang S., Ming Z., Chen H. (2020). Inhibition of keratinocyte necroptosis mediated by RIPK1/RIPK3/MLKL provides a protective effect against psoriatic inflammation. Cell Death Dis..

[161] Rathkey J.K., Zhao J., Liu Z., Chen Y., Yang J., Kondolf H.C., Benson B.L., Chirieleison S.M., Huang A.Y., Dubyak G.R. (2018). Chemical disruption of the pyroptotic pore-forming protein gasdermin D inhibits inflammatory cell death and sepsis. Sci. Immunol..

[162] Newton K., Dugger D.L., Maltzman A., Greve J.M., Hedehus M., Martin-McNulty B., Carano R.A., Cao T.C., van Bruggen N., Bernstein L. (2016). RIPK3 deficiency or catalytically inactive RIPK1 provides greater benefit than MLKL deficiency in mouse models of inflammation and tissue injury. Cell Death Differ..

[163] Weisel K., Berger S., Papp K., Maari C., Krueger J.G., Scott N., Tompson D., Wang S., Simeoni M., Bertin J. (2020). Response to Inhibition of Receptor-Interacting Protein Kinase 1 (RIPK1) in Active Plaque Psoriasis: A Randomized Placebo-Controlled Study. Clin. Pharmacol. Ther..

[164] Harris P.A., Berger S.B., Jeong J.U., Nagilla R., Bandyopadhyay D., Campobasso N., Capriotti C.A., Cox J.A., Dare L., Dong X. (2017). Discovery of a First-in-Class Receptor Interacting Protein 1 (RIP1) Kinase Specific Clinical Candidate (GSK2982772) for the Treatment of Inflammatory Diseases. J. Med. Chem..

[165] de Souza H.S., Fiocchi C. (2016). Immunopathogenesis of IBD: Current state of the art. Nat. Rev. Gastroenterol. Hepatol..

[166] Turner J.R. (2009). Intestinal mucosal barrier function in health and disease. Nat. Rev. Immunol..

[167] Kiesslich R., Duckworth C.A., Moussata D., Gloeckner A., Lim L.G., Goetz M., Pritchard D.M., Galle P.R., Neurath M.F., Watson A.J. (2012). Local barrier dysfunction identified by confocal laser endomicroscopy predicts relapse in inflammatory bowel disease. Gut.

[168] Buhner S., Buning C., Genschel J., Kling K., Herrmann D., Dignass A., Kuechler I., Krueger S., Schmidt H.H., Lochs H. (2006). Genetic basis for increased intestinal permeability in families with Crohn’s disease: Role of CARD15 3020insC mutation?. Gut.

[169] Cifaldi C., Chiriaco M., Di Matteo G., Di Cesare S., Alessia S., De Angelis P., Rea F., Angelino G., Pastore M., Ferradini V. (2017). Novel X-Linked Inhibitor of Apoptosis Mutation in Very Early-Onset Inflammatory Bowel Disease Child Successfully Treated with HLA-Haploidentical Hemapoietic Stem Cells Transplant after Removal of alphabeta(+) T and B Cells. Front. Immunol..

[170] Lekbua A., Ouahed J., O’Connell A.E., Kahn S.A., Goldsmith J.D., Imamura T., Duncan C.N., Kelsen J.R., Worthey E., Snapper S.B. (2019). Risk-factors Associated With Poor Outcomes in VEO-IBD Secondary to XIAP Deficiency: A Case Report and Literature Review. J. Pediatr. Gastroenterol. Nutr..

[171] Serra E.G., Schwerd T., Moutsianas L., Cavounidis A., Fachal L., Pandey S., Kammermeier J., Croft N.M., Posovszky C., Rodrigues A. (2020). Somatic mosaicism and common genetic variation contribute to the risk of very-early-onset inflammatory bowel disease. Nat. Commun..

[172] Girardelli M., Arrigo S., Barabino A., Loganes C., Morreale G., Crovella S., Tommasini A., Bianco A.M. (2015). The diagnostic challenge of very early-onset enterocolitis in an infant with XIAP deficiency. BMC Pediatr..

[173] Latour S., Aguilar C. (2015). XIAP deficiency syndrome in humans. Semin. Cell Dev. Biol..

[174] Uhlig H.H. (2013). Monogenic diseases associated with intestinal inflammation: Implications for the understanding of inflammatory bowel disease. Gut.

[175] Nielsen O.H., LaCasse E.C. (2017). How genetic testing can lead to targeted management of XIAP deficiency-related inflammatory bowel disease. Genet. Med..

[176] Stafford C.A., Lawlor K.E., Heim V.J., Bankovacki A., Bernardini J.P., Silke J., Nachbur U. (2018). IAPs Regulate Distinct Innate Immune Pathways to Co-ordinate the Response to Bacterial Peptidoglycans. Cell Rep..

[177] Negroni A., Stronati L., Pierdomenico M., Tirindelli D., Di Nardo G., Mancini V., Maiella G., Cucchiara S. (2009). Activation of NOD2-mediated intestinal pathway in a pediatric population with Crohn’s disease. Inflamm. Bowel Dis..

[178] Stronati L., Negroni A., Merola P., Pannone V., Borrelli O., Cirulli M., Annese V., Cucchiara S. (2008). Mucosal NOD2 expression and NF-kappaB activation in pediatric Crohn’s disease. Inflamm. Bowel Dis..

[179] Stronati L., Negroni A., Pierdomenico M., D’Ottavio C., Tirindelli D., Di Nardo G., Oliva S., Viola F., Cucchiara S. (2010). Altered expression of innate immunity genes in different intestinal sites of children with ulcerative colitis. Dig. Liver Dis..

[180] Chirieleison S.M., Marsh R.A., Kumar P., Rathkey J.K., Dubyak G.R., Abbott D.W. (2017). Nucleotide-binding oligomerization domain (NOD) signaling defects and cell death susceptibility cannot be uncoupled in X-linked inhibitor of apoptosis (XIAP)-driven inflammatory disease. J. Biol. Chem..

[181] Kaser A., Zeissig S., Blumberg R.S. (2010). Inflammatory bowel disease. Annu. Rev. Immunol..

[182] Peyrin-Biroulet L. (2010). Anti-TNF therapy in inflammatory bowel diseases: A huge review. Minerva Gastroenterol. Dietol..

[183] Liu Z., Kong F., Vallance J.E., Harmel-Laws E., Amarachintha S., Steinbrecher K.A., Rosen M.J., Bhattacharyya S. (2017). Activation of TGF-beta activated kinase 1 promotes colon mucosal pathogenesis in inflammatory bowel disease. Physiol. Rep..

[184] Nenci A., Becker C., Wullaert A., Gareus R., van Loo G., Danese S., Huth M., Nikolaev A., Neufert C., Madison B. (2007). Epithelial NEMO links innate immunity to chronic intestinal inflammation. Nature.

[185] Vlantis K., Wullaert A., Polykratis A., Kondylis V., Dannappel M., Schwarzer R., Welz P., Corona T., Walczak H., Weih F. (2016). NEMO Prevents RIP Kinase 1-Mediated Epithelial Cell Death and Chronic Intestinal Inflammation by NF-kappaB-Dependent and -Independent Functions. Immunity.

[186] Chae S., Eckmann L., Miyamoto Y., Pothoulakis C., Karin M., Kagnoff M.F. (2006). Epithelial cell I kappa B-kinase beta has an important protective role in Clostridium difficile toxin A-induced mucosal injury. J. Immunol..

[187] Eckmann L., Nebelsiek T., Fingerle A.A., Dann S.M., Mages J., Lang R., Robine S., Kagnoff M.F., Schmid R.M., Karin M. (2008). Opposing functions of IKKbeta during acute and chronic intestinal inflammation. Proc. Natl. Acad. Sci. USA.

[188] Kajino-Sakamoto R., Inagaki M., Lippert E., Akira S., Robine S., Matsumoto K., Jobin C., Ninomiya-Tsuji J. (2008). Enterocyte-derived TAK1 signaling prevents epithelium apoptosis and the development of ileitis and colitis. J. Immunol..

[189] Fish J.D., Duerst R.E., Gelfand E.W., Orange J.S., Bunin N. (2009). Challenges in the use of allogeneic hematopoietic SCT for ectodermal dysplasia with immune deficiency. Bone Marrow Transplant..

[190] Kawai T., Nishikomori R., Heike T. (2012). Diagnosis and treatment in anhidrotic ectodermal dysplasia with immunodeficiency. Allergol. Int..

[191] Pai S.Y., Levy O., Jabara H.H., Glickman J.N., Stoler-Barak L., Sachs J., Nurko S., Orange J.S., Geha R.S. (2008). Allogeneic transplantation successfully corrects immune defects, but not susceptibility to colitis, in a patient with nuclear factor-kappaB essential modulator deficiency. J. Allergy Clin. Immunol..

[192] Permaul P., Narla A., Hornick J.L., Pai S.Y. (2009). Allogeneic hematopoietic stem cell transplantation for X-linked ectodermal dysplasia and immunodeficiency: Case report and review of outcomes. Immunol. Res..

[193] Anderson C.A., Boucher G., Lees C.W., Franke A., D’Amato M., Taylor K.D., Lee J.C., Goyette P., Imielinski M., Latiano A. (2011). Meta-analysis identifies 29 additional ulcerative colitis risk loci, increasing the number of confirmed associations to 47. Nat. Genet..

[194] Vereecke L., Vieira-Silva S., Billiet T., van Es J.H., Mc Guire C., Slowicka K., Sze M., van den Born M., De Hertogh G., Clevers H. (2014). A20 controls intestinal homeostasis through cell-specific activities. Nat. Commun..

[195] Vereecke L., Sze M., Mc Guire C., Rogiers B., Chu Y., Schmidt-Supprian M., Pasparakis M., Beyaert R., van Loo G. (2010). Enterocyte-specific A20 deficiency sensitizes to tumor necrosis factor-induced toxicity and experimental colitis. J. Exp. Med..

[196] Kolodziej L.E., Lodolce J.P., Chang J.E., Schneider J.R., Grimm W.A., Bartulis S.J., Zhu X., Messer J.S., Murphy S.F., Reddy N. (2011). TNFAIP3 maintains intestinal barrier function and supports epithelial cell tight junctions. PLoS ONE.

[197] Rhee L., Murphy S.F., Kolodziej L.E., Grimm W.A., Weber C.R., Lodolce J.P., Chang J.E., Bartulis S.J., Messer J.S., Schneider J.R. (2012). Expression of TNFAIP3 in intestinal epithelial cells protects from DSS- but not TNBS-induced colitis. Am. J. Physiol. Gastrointest. Liver Physiol..

[198] Garcia-Carbonell R., Wong J., Kim J.Y., Close L.A., Boland B.S., Wong T.L., Harris P.A., Ho S.B., Das S., Ernst P.B. (2018). Elevated A20 promotes TNF-induced and RIPK1-dependent intestinal epithelial cell death. Proc. Natl. Acad. Sci. USA.

[199] Alvarez-Diaz S., Dillon C.P., Lalaoui N., Tanzer M.C., Rodriguez D.A., Lin A., Lebois M., Hakem R., Josefsson E.C., O’Reilly L.A. (2016). The Pseudokinase MLKL and the Kinase RIPK3 Have Distinct Roles in Autoimmune Disease Caused by Loss of Death-Receptor-Induced Apoptosis. Immunity.

[200] Zhang H., Zhou X., McQuade T., Li J., Chan F.K., Zhang J. (2011). Functional complementation between FADD and RIP1 in embryos and lymphocytes. Nature.

[201] Welz P.S., Wullaert A., Vlantis K., Kondylis V., Fernandez-Majada V., Ermolaeva M., Kirsch P., Sterner-Kock A., van Loo G., Pasparakis M. (2011). FADD prevents RIP3-mediated epithelial cell necrosis and chronic intestinal inflammation. Nature.

[202] Gunther C., Martini E., Wittkopf N., Amann K., Weigmann B., Neumann H., Waldner M.J., Hedrick S.M., Tenzer S., Neurath M.F. (2011). Caspase-8 regulates TNF-alpha-induced epithelial necroptosis and terminal ileitis. Nature.

[203] Stolzer I., Kaden-Volynets V., Ruder B., Letizia M., Bittel M., Rausch P., Basic M., Bleich A., Baines J.F., Neurath M.F. (2020). Environmental Microbial Factors Determine the Pattern of Inflammatory Lesions in a Murine Model of Crohn’s Disease-Like Inflammation. Inflamm. Bowel Dis..

[204] Schwarzer R., Jiao H., Wachsmuth L., Tresch A., Pasparakis M. (2020). FADD and Caspase-8 Regulate Gut Homeostasis and Inflammation by Controlling MLKL- and GSDMD-Mediated Death of Intestinal Epithelial Cells. Immunity.

[205] Wittkopf N., Gunther C., Martini E., He G., Amann K., He Y.W., Schuchmann M., Neurath M.F., Becker C. (2013). Cellular FLICE-like inhibitory protein secures intestinal epithelial cell survival and immune homeostasis by regulating caspase-8. Gastroenterology.

[206] Lehle A.S., Farin H.F., Marquardt B., Michels B.E., Magg T., Li Y., Liu Y., Ghalandary M., Lammens K., Hollizeck S. (2019). Intestinal Inflammation and Dysregulated Immunity in Patients With Inherited Caspase-8 Deficiency. Gastroenterology.

[207] Uchiyama Y., Kim C.A., Pastorino A.C., Ceroni J., Lima P.P., de Barros Dorna M., Honjo R.S., Bertola D., Hamanaka K., Fujita A. (2019). Primary immunodeficiency with chronic enteropathy and developmental delay in a boy arising from a novel homozygous RIPK1 variant. J. Hum. Genet..

[208] Dourmashkin R., Davies H., Wells C., Shah D., Price A., O’Morain J., Levi J. (1989). Epithelial patchy necrosis in Crohn’s disease. Hum. Pathol..

[209] Pierdomenico M., Negroni A., Stronati L., Vitali R., Prete E., Bertin J., Gough P.J., Aloi M., Cucchiara S. (2014). Necroptosis is active in children with inflammatory bowel disease and contributes to heighten intestinal inflammation. Am. J. Gastroenterol..

[210] Negroni A., Colantoni E., Pierdomenico M., Palone F., Costanzo M., Oliva S., Tiberti A., Cucchiara S., Stronati L. (2017). RIP3 AND pMLKL promote necroptosis-induced inflammation and alter membrane permeability in intestinal epithelial cells. Dig. Liver Dis..

[211] Wu T., Dai Y., Xue L., Sheng Y., Xu L., Xue Y. (2019). Expression of receptor interacting protein 3 and mixed lineage kinase domain-like protein-key proteins in necroptosis is upregulated in ulcerative colitis. Ann. Palliat. Med..

[212] Zhou M., He J., Shi Y., Liu X., Luo S., Cheng C., Ge W., Qu C., Du P., Chen Y. (2021). ABIN3 Negatively Regulates Necroptosis-induced Intestinal Inflammation Through Recruiting A20 and Restricting the Ubiquitination of RIPK3 in Inflammatory Bowel Disease. J. Crohns Colitis..

[213] Gobbetti T., Berger S.B., Fountain K., Slocombe T., Rowles A., Pearse G., Harada I., Bertin J., Haynes A.C., Beal A.M. (2020). Receptor-interacting protein 1 kinase inhibition therapeutically ameliorates experimental T cell-dependent colitis in mice. Cell Death Dis..

[214] Zhang J., Qin D., Yang Y.J., Hu G.Q., Qin X.X., Du C.T., Chen W. (2019). MLKL deficiency inhibits DSS-induced colitis independent of intestinal microbiota. Mol. Immunol..

[215] Zhao Q., Yu X., Li M., Liu Y., Han Y., Zhang X., Li X.M., Wu X., Qin J., Fang J. (2019). MLKL attenuates colon inflammation and colitis-tumorigenesis via suppression of inflammatory responses. Cancer Lett..

[216] Alvarez-Diaz S., Preaudet A., Samson A.L., Nguyen P.M., Fung K.Y., Garnham A.L., Alexander W.S., Strasser A., Ernst M., Putoczki T.L. (2020). Necroptosis is dispensable for the development of inflammation-associated or sporadic colon cancer in mice. Cell Death Differ..

[217] Lu H., Li H., Fan C., Qi Q., Yan Y., Wu Y., Feng C., Wu B., Gao Y., Zuo J. (2020). RIPK1 inhibitor ameliorates colitis by directly maintaining intestinal barrier homeostasis and regulating following IECs-immuno crosstalk. Biochem. Pharmacol..

